# High-Performing Young Musicians’ Playing-Related Pain. Results of a Large-Scale Study

**DOI:** 10.3389/fpsyg.2020.564736

**Published:** 2020-12-16

**Authors:** Heiner Gembris, Jonas Menze, Andreas Heye, Claudia Bullerjahn

**Affiliations:** ^1^Faculty of Cultural Studies, Institute for Research on Musical Ability, Paderborn University, Paderborn, Germany; ^2^Faculty of Linguistics and Literary Studies, Art and Music Education, Bielefeld University, Bielefeld, Germany; ^3^Department of Social Sciences and Cultural Studies, Institute of Musicology and Music Education, Justus Liebig University Giessen, Giessen, Germany

**Keywords:** music students, playing-related pain, music making, musical talent, music education, high performers, adolescents, musical practice

## Abstract

The present study examines the prevalence, localization, frequency, and intensity of playing-related pain (PRP) in a sample of high-performing young musicians. We also address coping behavior and communication about PRP between young musicians, teachers, parents, and other people, such as friends. The aim is to provide information on PRP among high-performing musicians in childhood and adolescence, which can serve as a basis for music education, practice, and prevention in the context of instrumental teaching and musicians’ health. The study is part of a large-scale study (*N* = 1,143) with highly musically gifted participants (age 9–24 years; *M* = 15.1; *SD* = 2.14, female = 62%) at the national level of the “Jugend musiziert” (youth making music) contest. For data analyses, we used descriptive statistics, correlations, Chi^2^-tests, principal component analysis, Kruskal–Wallis *H* tests, and multivariate regression. About three-quarters (76%) of the surveyed participants stated that they had experienced pain during or after playing their instrument. Female musicians were significantly more frequently affected (79%) than male musicians (71%). With increasing age, the prevalence of PRP rises from 71 percent (9–13 years) to 85 percent (18–24 years). Regarding localization of pain, results are in line with many other studies with musculoskeletal problems the most common. Furthermore, data show a clear relationship between the duration of practice and the prevalence of PRP. Our study found averages of 7:18 h/week, whereas mean values of the duration of practice vary considerably between different instruments. The variance in practice duration is very large within the different instruments. Thus, when researching PRP, it is necessary to consider both the differences between different groups of instruments in the average duration of practice as well as the very large inter-individual variation in the duration of practice within a given instrument group. While just over half of the young musicians (56%) felt they had been taken seriously, 32 percent felt that their complaints were not completely taken seriously, and 12 percent did not feel taken seriously at all. Therefore, it is necessary to improve communication and information about PRP to prevent PRP and counteract existing complaints.

## Introduction

The present study is a part of a comprehensive large-scale study of adolescent, highly talented musicians participating in the 2017 national contest “Jugend musiziert” (youth making music) in Germany (Gembris and Bullerjahn, 2018; Bullerjahn et al., 2020). This annual contest has existed for more than 50 years and is the largest and most important contest for young musicians in Germany. The participants on the national level competition have successfully passed the regional and federal state levels beforehand and are commonly regarded as highly gifted and belonging to the national elite of young musicians. In 2017, the national competition “Jugend musiziert,” which was carried out in the city of Paderborn (Germany), was announced for the following instruments/categories: piano, harp, voice, drum set (pop), guitar (pop), string ensemble, wind ensemble, chamber music for accordions, and “Neue Musik” (New Music; i.e., avant-garde music of the 20th century/contemporary music) (Deutscher Musikrat, 2017, p. 1). The general objective of this large-scale study on the participants of the national contest “Jugend musiziert” is to collect basic information on the personality and sociocultural environment of the competition participants, their motivations, amount of practice, musical attitudes, etc. (see Bullerjahn et al., 2020). The subject of the present paper is playing-related pain (PRP) among high-performing young musicians, a topic that has rarely been investigated and which we aim to explore using the example of participants in the “Jugend musiziert” national contest.

### Playing-Related Pain (PRP) Among Young Musicians

It is well-known that professional musicians often suffer from physical complaints and pain when playing. Numerous studies demonstrate that between 60 and 90 percent of professional musicians suffer from pain of the musculoskeletal system (e.g., Fishbein et al., 1988; Blum, 1995; Spahn and Möller, 2011; Ackermann et al., 2012; Kenny and Ackermann, 2013; Gembris et al., 2018). In contrast, pain in the context of music-making by children and adolescents is hardly addressed in musicians’ medicine and instrumental pedagogy. Rather incidental results from instrumental pedagogical studies provide indications that pain in connection with the playing of instruments by children and adolescents could be a problem.

According to reviews of literature (Spahn, 2015, p. 131; Spahn, 2011, pp. 11 f.), two-thirds of young instrumentalists (children and adolescents) have experiences with PRP. Various studies from the field of musicians’ medicine indicate that PRP in children and adolescents is not uncommon (e.g., Fry and Rowley, 1989; Samsel et al., 2005; McKechnie and Jacobs, 2011; Ranelli et al., 2011; Spahn, 2011; Gembris and Ebinger, 2017). However, data concerning the prevalence of PRP in young musicians (10–18 years) vary considerably between 33 percent up to 96 percent (e.g., Bruno et al., 2008: 33%; Nawrocka et al., 2014: 88%; Harnischmacher, 1993: 96%; for an overview see Gembris and Ebinger, 2017).

The differences in these results can have various reasons, for example differences in age samples, instrument groups or degrees of professionalism and duration of instrumental play. There are also other factors such as the different methods of assessing the types of PRP, their localization, intensity, duration, time periods of occurrence of PRP, and different rating scales for recording the duration of individual practice and practice strategies, etc. (see Stanhope et al., 2019 for a comprehensive overview).

It is assumed that the risk of developing PRP depends on a variety of interacting factors. A first important factor in the etiology of PRP is the kind of instrument played. Some studies found the highest risks with strings and piano (e.g., Nawrocka et al., 2014; Ioannou and Altenmüller, 2015). Others observed the highest risks for wind instrumentalists (e.g., Stanek et al., 2017). A third group of studies reported no differences (Robitaille et al., 2018). Inconsistencies in the results could, for example, be caused by differences in methodology, samples, or duration of instrumental playing.

A second risk factor is the duration of practice, which can vary considerably from instrument to instrument (see Jørgensen, 1997, pp. 132 f.). Piano or violin are practiced for much longer in a single practice session than wind instruments such as oboe, horn, trumpet, or the singing voice. One reason is that the embouchure and lip tension of a wind instrument and the vocal chords get tired faster and are not as resilient as the strings of a violin or piano. The appraisal of whether someone practices a lot or less should therefore be considered with regard to the nature of the instrument and its specific playing requirements.

Kaczmarek (2012) carried out a study with young musicians, differing in the level of expertise. One group of these musicians comprised high-performing “experts,” who were enrolled in courses at music universities with special programs for particularly gifted students. The other group consisted of “non-expert” students of municipal music schools. The experts had played their instrument significantly longer and had spent significantly more time practicing. Overall, 75 percent of adolescents (*N* = 120; average age *M* = 15; *SD* = 1.98) regularly reported PRP – mainly musculoskeletal complaints – both during and after playing (Kaczmarek, 2012, p. 280). The experts tended to have a higher prevalence of PRP (78%) compared to the non-expert music students (69%), but the differences were not significant. The main complaint areas were the back (39%), the neck and shoulder area (43%), and the arm and hand area (28%). In another sample of 225 young “pre-professional” students aged 10–18 years from special music schools, Nawrocka et al. (2014) found an even higher prevalence of PRP of 88 percent. These high prevalence values correspond to those of professional musicians who have often played their instrument for many hours a day for decades (e.g., Gembris et al., 2018). The question arises as to why young musicians can achieve a similarly high prevalence of PRP as professional musicians.

A further aspect of duration of practice is the number of years an instrument is played. According to the study by Nawrocka et al. (2014), the prevalence of PRP seems to increase with each year of practice, at least among students who practice intensively. Gembris and Ebinger (2017) found, in a sample of students of a music school, that the prevalence of PRP rises significantly with a playing time or teaching period of more than 8 years.

A third risk factor of importance are practice habits. Sudden changes in practice habits (e.g., increased practice time) may also increase PRP (Ioannou et al., 2018; Robitaille et al., 2018). Furthermore, practice without rest and without warm-up/cool-down is related to an increase in PRP (Ioannou and Altenmüller, 2015; Ling et al., 2018).

As a fourth risk factor, gender differences may also play a role. Generally, female musicians are more frequently affected than male musicians (e.g., Fry et al., 1988; Lockwood, 1988; Fry and Rowley, 1989; Britsch, 2005; Bruno et al., 2008; Ranelli et al., 2008; Nawrocka et al., 2014; Ioannou and Altenmüller, 2015). However, results are inconsistent, and studies on PRP in high-performing children and adolescents are especially scarce (e.g., Heye, 2017, 2019).

Beside these relatively often examined risk factors, further factors were subject of scientific studies. Ballenberger et al. (2018) investigated the risk factors for musculoskeletal health complaints (MHC) in a prospective longitudinal study in a sample of music students compared to non-musical students. They found that risk factors for MHC “included being a music student, previous pain, reduced physical functioning, stress symptoms, reduced emotional functioning, and mechanosensitivity” (p. 166). The authors conclude: “The variables we identified relate to physical, occupational, psychosocial aspects, and pain and confirm the idea that MHC may be the result of a complex interaction of a variety of contributing mechanisms.” (p. 172).

Evidence for the influence of psychological and social factors in the etiology of PRP comes also from a study conducted by Heye (2019). He surveyed musically gifted adolescents in relation to chronic stress. The participants reported a variety of physical and psychological stress symptoms (e.g., exhaustion or tension headaches). These stressors are clearly attributable to the interaction of musical demands (e.g., practicing), their own performance expectations, and to persistent social conflicts with significant others at school or at home. Adolescents often lack adequate coping strategies to deal with chronic stress and time pressure, which is why it is important for parents or peers to intervene in a supportive manner.

In a recent review of musculoskeletal complaints in musicians, Stanhope et al. (2019) have compiled a detailed list of perceived risks or aggravating factors, which have been investigated in previous studies. In addition to the risk factors mentioned here, further factors are specified, some of which appear to be very individual (e.g., technique flaws, playing when physically exhausted) or do not appear to be music-specific or instrument-specific (e.g., emotional problems, lack of social support; see pp. 303 f.).

### Communication About PRP Among Students, Teachers, and Parents

Communication about PRP among students, teachers, and parents has rarely been investigated (e.g., Birkedahl, 1989; Britsch, 2005; Ackermann and Driscoll, 2013). Ackermann and Driscoll (2013) explored the attitudes and practices of parents (*N* = 23) of teenage musicians (14–17 years) in regard to health issues related to playing an instrument (mainly piano, drums, and flute). They found that almost all parents “strongly agreed or agreed that their child’s physical health was important for playing their instrument well, that their child’s ability to cope with performance anxiety was important to playing their instrument well, that their child’s understanding of how the body works was important to play their instrument well, and that their child should learn about how his/her body works when training to become a skilled musician.” (pp. 25 f.) These parents also judged the item “I consider that pain associated with playing an instrument is normal.” Most of the parents (70%) disagreed with this item, 17 percent agreed, and 13 percent were neutral. Interestingly, there were striking differences in the responses given by parents whose children attended an academically selective public high school and those whose children attended a public high school specialized on music. All parents of the children at the academically selective high school disagreed with this item, but 29 percent of the parents of children at the specialized music high school agreed and further 21 percent were neutral. The authors suppose “that the parents of children at the musically selective school, where presumably the playing is taken more seriously, would be more likely to accept their child experiencing pain, perhaps because pain is more accepted by the parents as a normal part of playing” (Ackermann and Driscoll, 2013, p. 26). With regard to the main limitation of the study (convenience sample of relatively few parents; *N* = 23) the authors believe that it is “impossible to be confident to what extent the results reflect the larger parental population from which the participants were drawn” (Ackermann and Driscoll, 2013, p. 27).

Gembris and Ebinger (2017) investigated the prevalence of PRP among young instrumentalists (children and adolescents), risk factors, and coping strategies. To the best of our knowledge, this study was the first to examine communication about PRP between instrumental students, teachers, and parents. The authors developed three different forms of standardized questionnaires, specifically designed for (a) students, (b) parents, and (c) teachers, which were distributed by the music school. The participants (*N* = 800) in this study consisted of (a) students of a communal music school (*n* = 399; age 7–23 years; *M* = 13; *SD* = 2.67), (b) their parents (*n* = 367), and (c) instrumental teachers (*n* = 34).

Half of the students (51%) indicated PRP during and/or after playing. No gender differences were observed. Of those who reported PRP, 10 percent suffered from severe pain. As in many other studies, musculoskeletal complaints were the main focus. The weekly practice time was also very different in this study and varied between 10 min and 10 h, the mean value was only 2.2 h/week (*SD* = 1.5; Gembris and Ebinger, 2017, pp. 137 f.). Concerning the communication of PRP, the authors found that 71 percent had spoken about PRP with their mother, 41 percent with their teacher, and 33 percent with their father. 15 percent had spoken with other people and 14 percent with their friends (multiple answers had been possible). Almost one third (32%) of the parents and 56 percent of the teachers had previously noticed that their children resp. students had PRP. Teachers underestimated the prevalence of PRP: most teachers (89%) estimated that only 1–20 percent of the students may experience PRP. The localization of pain was often misjudged by the teachers.

One very important question is whether students who report PRP feel that their complaints about PRP are taken seriously. About half of the children answered “yes” (51%). Almost one fifth (19%) did not feel that their pain was taken seriously, and about a third (31%) only partially. When these two groups are considered together, about half of those who talked to parents or teachers about PRP did not really feel taken seriously. These results demonstrate that there exists a problem in communication about PRP, which needs to be studied more closely.

### Aims and Research Questions

This paper specifically explores the prevalence and characteristics of PRP in high-performing young musicians who participated in the national competition “Jugend musiziert” and how they cope with it. Thus, we will deal with

(a) the prevalence of PRP among high-performing young musicians,
(b) the relationship between PRP and personal variables such as gender, age, and amount of practice,
(c) the localization, frequency, and intensity of PRP for different instruments or groups of instruments,
(d) the coping behavior with PRP in young high-performing musicians,
(e) the communication about PRP between the young musicians, their parents, and teachers and the feeling to be taken seriously with the complaints.

Based on research findings on PRP, we expect that female competitors suffer more from PRP than male competitors, that violinists and pianists have greater practice intensity than other instrument groups and therefore have a higher risk for PRP, and that PRP is most prevalent in the back and neck area.

## Materials and Methods

### Questionnaire and Procedure

For the examination of the competition participants, we developed a standardized paper-pencil questionnaire (17 pages, including some open questions), which covers a broad range of aspects, e.g., the instrument played, personal experiences with the “Jugend musiziert” contest, sociocultural variables, personality, musical training and practice, motivation, musical preferences, and support from the family, as well as questions concerning PRP and other health aspects (for more detailed information see Gembris and Bullerjahn, 2018, 2019; Bullerjahn et al., 2020). Personality factors were recorded using the BFI-10 inventory (Rammstedt et al., 2012). The questions related to PRP are documented in the Supplementary Section “Supplementary Excerpt from the Questionnaire.”

A few weeks before the contest started, all participants and their parents received a letter which informed them about the research project and asked them to participate. They were informed that participation was voluntary, anonymous, and independent of the contest. During the 6 days of the competition, approx. 2,260 participants (of a total of 2,732 registered cases that includes double participation, if someone participated in the solo as well as in the ensemble competition or as accompanist) could be contacted personally when they enrolled at the central registration desk for the national contest. They were asked to fill in the questionnaire and return it in the following days.

### Data Analyses

According to the explorative character of the study, we used descriptive statistics to describe the general characteristics of the data. The central tendencies and distribution of data were assessed by the mean or median and standard deviation. The range resp. distribution of the data is visualized in graphic diagrams. To investigate whether PRP is more prevalent in young musicians who practice more on their instrument compared to those who practice less on the same instrument, we calculated the median of the weekly practice time for each instrument and assigned the young musicians into groups of those who practiced a lot (practice time above median) and those who practiced little (practice time is below or meets median).Chi^2^-tests or *t*-tests were calculated to identify possible group differences (gender, age groups, specific instrument groups) concerning the prevalence of PRP, the intensity of complaints, practice habits, and other aspects. Possible linear relationships between two variables (e.g., well-being and intensity of PRP) have been tested using bivariate correlations.

We conducted a principal component factor analysis to investigate the correlation of PRP in different body regions. Additionally, a multivariate regression model was used to predict the frequency and intensity of PRP (stepwise linear regression).

Given the fact that the individual instruments are represented in different proportions in the data set, they were condensed into larger instrument groups for further analysis (percussion instruments, brass instruments, woodwind instruments, keyboard instruments, plucked string instruments, bowed string instruments, voice). Finally, a Kruskal–Wallis *H* test was conducted to examine the differences on intensity of PRP between different instrument groups.

Data analysis was carried out using the IBM Statistical Packages for the Social Sciences (SPSS, Version 26).

## Results

### Description of the Sample, Pain Prevalence

Of approx. 2,260 questionnaires handed out, a total number of *N* = 1,143 has been returned (rate of return = approx. 50%). The age of the young musicians ranged from 9 to 24 years (*M* = 15.1; *SD* = 2.14; age distribution can be found in Supplementary Figure 1), nearly two-thirds (62%, *n* = 692) were female, a little more than one-third (38%, *n* = 427) was male. There were no gender differences regarding the age distribution. The majority (70%) competed in the ensemble contests (strings, winds, accordion, “Neue Musik”), a smaller part (28%) participated in the solo contests (piano, harp, voice, drum set, and/or guitar), 1 percent competed in both the ensemble contest and the solo contest, and 2 percent were accompanists.

About three-quarters (76%; *n* = 850) of the participants stated that they had experienced pain during or after playing their instrument. Female musicians were significantly more often affected (79%) than male musicians (71%; Chi^2^ = 9.06; *p* = 0.003; Cramer’s *V* = 0.091; *n* = 1,105). This confirms our expectation as well as the results of most studies that female musicians have a higher prevalence of PRP.

The prevalence of PRP differs significantly between age groups (Chi^2^ = 14.84; *p* = 0.002; Cramer’s *V* = 0.116). Adolescents are more frequently affected than children. The proportion of competition participants with PRP increases from 71 percent in the group of the youngest participants (9–13 years) to 85 percent in the group of the oldest participants (18–24 years; see Table 1). Among those who claimed to suffer from PRP, about one-fifth (22%) said they also had pain that had nothing to do with making music. Within this subgroup there was a total of *n* = 204 complaints that were mentioned (multiple nominations were possible). The most frequently mentioned complaints were musculoskeletal complaints (53% in total) and headaches (23%) (for a detailed overview see Table 2).

**TABLE 1:** Prevalence of PRP in different age groups.

**Age groups**	**9–13 years (*n* = 290)**	**14–15 years (*n* = 356)**	**16–17 years (*n* = 315)**	**18–24 years (*n* = 148)**
Prevalence of PRP	71%	73%	80%	85%

*Chi^2^ = 14.84; p = 0.002; Cramer’s V = 0.116.*

**TABLE 2:** Physical complaints not connected to instrumental playing.

**Area of complaints**	***n***	**%**
Back	41	20
Neck/shoulder	17	8
Knee	29	14
Other musculoskeletal complaints	22	11
Headache	46	23
Abdominal pain	9	4
Other	40	20
Overall	204	100

### Instruments, Time of Weekly Practice, and PRP

On average, the participants indicated a weekly practice time of 7 h and 18 min (*SD* = 6:18; *Mdn* = 05:55; *n* = 1,070).^1^ No gender differences could be observed in the average practice time. Table 3 gives an overview of the weekly practice time for different instruments as estimated by the participants. Players of piano, violin, and harp show the highest means of practice time with almost 10–11 h per week. On the other hand, for players of wind instruments such as saxophone and tuba and for singers the mean of the weekly practice time amounts to 3 to almost 4 h, just one-third of the practice time of the piano, violin, or harp.

**TABLE 3:** Weekly practice time for different instruments as estimated by the participants.

**Instrument**	***n***	**min**	**max**	***M***	***SD***	***M* diff.**	***p***	***d***	***Mdn***
Piano	208	00:30	38:30	10:50	07:48	+03:32	< 0.001	0.50	09:00
Violin	131	01:00	38:30	09:57	06:45	+02:39	< 0.001	0.41	09:00
Harp	34	00:40	31:30	09:20	06:48	+02:02	n. s.	–	06:45
Viola	27	01:30	21:00	08:45	04:26	+01:27	n. s.	–	08:00
Cello	79	00:30	28:00	08:41	06:03	+01:23	0.046	0.22	07:00
Trumpet	54	01:27	35:00	06:49	06:26	−00:29	n. s.	–	04:30
Guitar	14	01:30	17:30	06:36	05:06	−00:42	n. s.	–	05:56
Trombone	21	00:45	17:30	05:57	04:30	−01:21	n. s.	–	04:30
Bassoon	30	01:07	21:00	05:56	04:39	−01:22	n. s.	–	04:45
Horn	45	00:30	28:00	05:52	05:58	−01:26	n. s.	–	03:45
Accordion	37	01:00	14:00	05:50	03:18	−01:28	0.011	0.29	06:00
Oboe	21	01:40	15:00	05:32	03:23	−01:46	0.027	0.35	05:00
Clarinet	70	00:30	21:00	05:15	04:03	−02:03	< 0.001	0.39	04:07
Percussion	33	00:30	21:00	05:13	04:47	−02:05	0.018	0.37	04:00
Flute	144	00:30	28:00	04:46	04:28	−02:32	< 0.001	0.47	03:00
Voice	66	00:30	21:00	03:48	03:31	− 03:30	< 0.001	0.69	03:00
Tuba	25	00:30	10:30	03:24	02:14	−03:54	< 0.001	0.83	03:22
Saxophone	31	00:34	10:30	03:05	02:30	−04:13	< 0.001	0.88	02:00

*The table gives an overview of practice time in hours per week for different instruments: number of cases (n), minimum (min), maximum (max), mean (M), standard deviation (SD), difference of the instrument-specific mean (M diff.) from the general mean of the weekly practice time in the subsample of all instruments represented here (M = 07:18; SD = 6:18; n = 1,070), significance of these differences (p; t-test in single sample), effect size (Cohen’s d) and median (Mdn).*

The practice time for the different instruments is not normally distributed, but shows very different forms of distribution. Supplementary Figures 2–20 show the distributions of practice time for the entire sample and for the individual instruments. Overall, the type of distribution tends to be more or less right-skewed, both for the overall sample and for the individual instruments. Therefore, the median seems to be more appropriate for describing the central tendency than the mean. As shown in Table 3, the median of practice time is clearly below the mean value in the majority of cases. With regard to the evaluation of the distributions of the individual instruments, it should be noted that the instruments are represented differently in terms of their number. Piano (*n* = 208), flute (*n* = 144), and violin (*n* = 131) are more frequently represented than others (e.g., cello, *n* = 79; guitar, *n* = 14).

A second important observation is the high variance in weekly practice time within individual instruments, which can be seen in the distribution of practice time (see Supplementary Figures 3–20). This is reflected in the high standard deviations and is particularly evident in the minima and maxima (see Table 3). For example, the weekly practice time for piano and violin varies from 30 min to more than 38 h. The considerable variation in weekly practice time, which can be observed in all instruments, is striking. It is noticeable that in practically all instruments on the right side of the distribution there are single or multiple cases that practice comparatively long. For practically all instruments, there are cases, which exceed the average or median practice time of the respective instrument by a factor of three or more. It seems rather unlikely that these cases of intensive practice are merely outliers, as they occur with all instruments. Nor can they be attributed to the unreliability of smaller samples because they also occur in larger samples. We have examined those young musicians, who show such a high degree of overtime, in a short digression of their own (see section “Characteristics and Personality Traits of Participants With an Outstanding High Amount of Practice”).

Table 4 shows the prevalence of PRP for the respective instruments in total as well as a comparison between those, who practice a lot, and those, who practice little.

**TABLE 4:** Prevalence of PRP for different instruments and practicing efforts.

				**Percent of PRP**			

	**Total**	**Overall**		**Low amount of practice**		**High amount of practice**	
	***n***	***n***	**%**	***n***	**%**	***n***	**%**
Viola	27	26	96	13	93	13	100
Oboe	21	20	95	10	91	10	100
Clarinet	69	61	88	30	83	31	94
Accordion	37	32	87	16	84	16	89
Harp	34	29	85	14	82	15	88
Violin	131	111	85	56	80	55	90
Cello	76	64	84	32	76	32	94
Bassoon	29	24	83	13	87	11	79
Saxophone	31	25	81	13	81	12	80
Trombone	20	15	75	8	67	7	88
Percussion	33	24	73	12	63	12	86
Horn	44	32	73	14	64	18	82
Flute	142	100	70	46	64	54	77
Piano	204	143	70	72	66	71	76
Trumpet	53	34	64	17	63	17	65
Tuba	25	15	60	5	39	10	83
Voice	64	38	59	19	54	19	66
Guitar	14	8	57	3	43	5	71

*The percentages in the “Low/High amount of practice” columns refer to the percentage of those who reported suffering from PRP at the particular practice frequency on the particular instrument. The data set was split by median. Individuals whose practice time exactly meets the median have been assigned to the group of those with a low amount of practice. For this reason, the total case numbers in the two columns may differ.*

The overall prevalence of PRP varies substantially between different instruments. It ranges from 57 percent for the guitar as the relatively lowest value to 95 percent (oboe) and 96 percent (viola) as the highest values.

If we look separately at those, who practice a lot, and those, who practice less, we again find differences in the prevalence of PRP for each instrument. For some instruments, the differences are larger (>10 percentage points, e.g., cello), for some smaller (<10 percentage points, e.g., harp), in two cases (saxophone, trumpet) almost equal. For most instruments, those, who practice a lot, show a prevalence of PRP, which is 10 (e.g., violin, piano) to 28 percentage points (guitar) higher than prevalence of PRP concerning those, who practice less.

For viola, oboe, accordion, harp, saxophone, and trumpet, the difference in the prevalence of PRP between those, who practice a lot, and those, who practice little, is less than 10 percentage points. For most of these instruments, however, it should be noted that the overall prevalence of PRP is already high (between 81 and 96%, with only the trumpet showing a comparatively low overall prevalence of 64%). In the case of the viola and the oboe, all of those, who practice these instruments a lot (100%), report experience with PRP.

Saxophone and bassoon are exceptions. Saxophonists, who practice little, indicate an almost identical (81% vs. 80%) prevalence of PRP compared with those who practice a lot. In the case of the bassoon, the PRP prevalence is clearly higher for those, who practice little (87% vs. 79%).

Our expectation, that violinists and pianists have the highest prevalence of PRP was only partially confirmed. According to our results, the prevalence is highest for viola (96%) and oboe (95%); this is about 25 percentage points higher than for the piano (70%).

### Localization, Frequency, Intensity, and Duration of PRP

Of those participants reporting PRP, neck and shoulder area (69%), wrist (56%), back (55%), arms (54%), and fingers (51%) are the most frequently mentioned parts of the body, in which PRP occurs (see Table 5). This finding tends to agree with our expectation that the back and the neck and shoulder area are most commonly affected by PRP. Further descriptive analysis shows that in 9 out of 10 cases, PRP does not relate to a single body area, but to several body areas simultaneously. On average, four body areas are affected simultaneously (*n* = 828; *M* = 4.1; *SD* = 2.09; *min* = 1; *max* = 9).

**TABLE 5:** Localization and frequency of PRP.

**Localization of pain**	**Share of PRP**		**Frequency of those with PRP**		
	**Of those with PRP (*n* = 837)**	**Of all participants (*n* = 1,110)**	**Seldom**	**Sometimes/often**	**Always**
Neck and shoulder area	69%	52%	39%	56%	5%
Wrists	56%	42%	60%	38%	2%
Back	55%	42%	41%	54%	5%
Arms	54%	40%	57%	41%	2%
Fingers	51%	39%	51%	45%	4%
Hands	48%	36%	57%	41%	2%
Mouth/lips	37%	28%	47%	52%	1%
Head	23%	17%	69%	30%	1%
Legs	14%	11%	71%	26%	3%

A principal component factor analysis was conducted including nine variables indicating the body parts, where PRP can occur. Analysis extracted three factors with eigenvalues equal to or greater than 1.0. Varimax rotation of the factors yielded the factor structure given in Table 6.

**TABLE 6:** Results of the factor analysis: eigenvalues and explained variance.

**Factor**	**Eigenvalue**	**% of variance**	**Cumulative%**
1	2.71	30.09	30.09
2	1.41	15.69	45.77
3	1.21	13.42	59.20

The calculation of the Kaiser–Meyer–Olkin (KMO) criterion for sample adequacy analysis yielded a value of 0.705, which is considered acceptable for factor analysis. The first factor includes manual functions of hands, fingers, wrists, and arms and accounts for 30 percent of variance. The second factor accounts for 16 percent of variance and addresses the upper body area (back, neck and shoulder area, head). A third factor explains 13 percent of variance and includes the mouth/lips, but also the legs (see Table 7). The total explained variance sums up to 59 percent. Only the first two factors were transformed into the scales “upper limbs” (hands, fingers, wrists, and arms) and “upper body” (back, neck and shoulder area, head) and used for further analysis. While the scale “upper limbs” has an acceptable reliability (Cronbach’s α = 0.723) and the scale “upper body” shows almost acceptable values (Cronbach’s α = 0.687), the reliability of the third scale consisting of mouth/lips and legs is not satisfactory (Cronbach’s α = 0.344). For this reason, the body areas mouth/lips and legs are considered separately. There are significant differences between instrument groups on all scales and items (“upper limbs”: Chi^2^ = 56.25, *df* = 6, *p* < 0.001; “upper body”: Chi^2^ = 53.71; *df* = 6, *p* < 0.001; mouth/lips: Chi^2^ = 340.62, *df* = 6, *p* < 0.001; legs: Chi^2^ = 23.68, *df* = 6, *p* = 0.001).

**TABLE 7:** Results of the factor analysis for the body parts of the PRP (*n* = 549).

**Items**	**Upper limbs**	**Upper body**	**Third factor (not interpreted)**
Hands	**0.821**	0.099	0.106
Fingers	**0.698**	−0.076	0.080
Wrists	**0.669**	0.106	0.005
Arms	**0.651**	0.392	0.026
Back	0.084	**0.855**	0.009
Neck and shoulder area	0.142	**0.832**	−0.016
Head	0.027	**0.523**	0.519
Mouth/lips	−0.050	−0.163	**0.778**
Legs	0.206	0.135	**0.637**
Cronbach’s α	0.723	0.687	0.344
Variance explained (%)	30.09	15.69	13.42

*Extraction method: principal component analysis (PCA). Rotation method: Varimax with Kaiser-Normalization. KMO: 0.705, Bartlett: p < 0.001. Bold values indicate the highest factor loading for each item.*

More details on significant differences concerning scales (and items, respectively) and instrument groups are summarized in Table 8. Supplementary Figure 21 shows that percussionists, bowed strings, and players of keyboard instruments experience pain most frequently in fingers, arms, hands, and wrists (scale “upper limbs”). In contrast to that, PRP of the body areas of the scale “upper back” (back, head, neck, and shoulder area) are similarly evenly distributed between the instrument groups (see Supplementary Figure 22). Furthermore, brass and woodwind players as well as singers experience pain mostly in mouth and lips (see Supplementary Figure 23) while pain in the legs is most frequently experienced by percussionists and singers (see Supplementary Figure 24).

**TABLE 8:** Kruskal–Wallis *H* test on differences in frequency of PRP between instrument groups.

**Scales and single items**	**Instrument groups**	**Chi^2^**	***z*-values**	**Sig.**
Upper limbs (*n* = 549)	Voice vs. woodwinds	145.96	3.986	*p* = 0.001
	Voices vs. plucked string instruments	167.65	3.290	*p* = 0.021
	Voices vs. keyboard instruments	194.92	5.178	*p* < 0.001
	Voices vs. bowed string instruments	217.79	5.862	*p* < 0.001
	Voices vs. percussion instruments	280.51	4.439	*p* < 0.001
	Brass instruments vs. keyboard instruments	–79.35	–3.329	*p* = 0.018
	Brass instruments vs. bowed string instruments	–102.22	–4.433	*p* < 0.001
	Woodwinds vs. bowed string instruments	–71.84	–3.983	*p* = 001
Upper body (*n* = 549)	Woodwinds vs. bowed string instruments	–119.53	–6.627	*p* < 0.001
	Brass instruments vs. bowed string instruments	–119.21	–5.169	*p* < 0.001
	Keyboard instruments vs. bowed string instruments	63.63	3.177	*p* = 0.031
Mouth/lips (*n* = 625)	Percussion instruments vs. woodwinds	270.91	4.795	*p* < 0.001
	Percussion instruments vs. brass instruments	287.80	4.964	*p* < 0.001
	Bowed string instruments vs. woodwinds	255.33	14.360	*p* < 0.001
	Bowed string instruments vs. brass instruments	272.22	12.352	*p* < 0.001
	Plucked string instruments vs. woodwinds	229.41	5.771	*p* < 0.001
	Plucked string instruments vs. brass instruments	246.30	5.888	*p* < 0.001
	Keyboard instruments vs. woodwinds	224.22	11.917	*p* < 0.001
	Keyboard instruments vs. brass instruments	241.11	10.538	*p* < 0.001
	Voice vs. woodwinds	196.93	5.296	*p* < 0.001
	Voice vs. brass instruments	213.83	5.428	*p* < 0.001
Legs (*n* = 612)	Keyboard instruments vs. percussion instruments	156.25	4.589	*p* < 0.001
	Plucked string instruments vs. percussion instruments	156.10	3.836	*p* = 0.003
	Bowed string instruments vs. percussion instruments	137.42	4.071	*p* = 0.001
	Woodwinds vs. percussion instruments	–134.38	–4.010	*p* = 0.001
	Brass instruments vs. percussion instruments	–130.38	–3.701	*p* = 0.005

The average of pain intensity is *M* = 2.5 (*n* = 769; *SD* = 1.11; *min* = 1; *max* = 6). Table 9 describes the intensity of pain separately for those who practice a lot and those who practice less, as well as for the entire sub-sample of musicians who reported PRP. The highest pain intensity (scale points 5 and 6) is indicated by only a few (3 and 1% of those who practice less, and 5 and 1% of those who practice a lot). The differences in pain intensity between those who practice a lot and those who practice less are not very large. To simplify the overview somewhat, the six levels of the scale can be summarized in three groups: (a) mild pain (scale points 1 and 2), (b) moderate pain (scale points 3 and 4) and (c) severe pain (scale points 5 and 6).

**TABLE 9:** Intensity of reported pain (*N* = 709).

	**Intensity of PRP**					

	**1**	**2**	**3**	**4**	**5**	**6**
	**Mild pain**		**Moderate pain**		**Severe pain**	
Low amount of practice (*n* = 342)	21%	38%	25%	12%	3%	1%
High amount of practice (*n* = 367)	18%	36%	29%	11%	5%	1%
Total	20%	37%	27%	11%	4%	1%

Significantly more than half (57%) of those reporting PRP indicated only mild pain. Those who practice a lot and those who practice less differed only a few percentage points. The group of those reporting moderate pain comprises 39 percent of the sub-sample with pain (the deviation from the calculated sum of the individual columns of 38% results from the rounding of the decimal places). Again, differences between those who practice a lot and those who practice little are small and also amount to only a few percentage points. Only a small group of a total of 5 percent indicate severe pain. Overall, it can be said that PRP is weak or moderate in the vast majority (95%) of cases. Contrary to assumptions, there are hardly any differences in the intensity of pain between those who practice a lot and those who practice little, although there is a very slight tendency for those who practice a lot to report stronger pain, but the differences are only between two or three percentage points.

**FIGURE 1:**
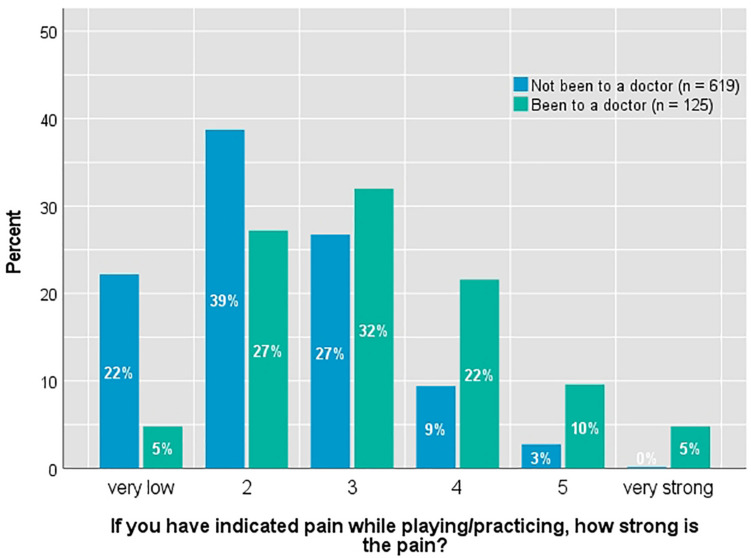
Distribution of pain intensity in differentiation according to the visit of a physician.

When further exploring the frequency of PRP, the individual highest value indicated when the individual body parts were queried was used as an indicator for the frequency of PRP in general. While female participants reported a higher frequency of PRP (*M* = 2.3; *SD* = 0.92) than male participants (*M* = 2.0; *SD* = 0.91; *t* = 5.08; *df* = 606.226; *p* < 0.001; *d* = 0.370), they did not differ in the intensity of pain. 22 percent reported suffering from pain not related to instrumental playing. They also reported a higher frequency of PRP (*M* = 2.5; *SD* = 0.89) than those not suffering from other pain (*M* = 2.1; *SD* = 0.93; *t* = −4.14; *df* = 780; *p* < 0.001; *d* = 0.362) and a higher intensity of PRP (*M* = 2.7; *SD* = 1.16) than those not suffering from other pain (*M* = 2.4; *SD* = 1.07; *t* = −3.02; *df* = 713; *p* = 0.003; *d* = 0.270). Pain intensity is also significantly higher (*t* = −7.45; *df* = 161.073; *p* < 0.001; *d* = 0.771) in the group of musicians who consulted a medical practitioner because of their pain (*n* = 125; *M* = 3.2; *SD* = 1.21) compared to the group of musicians who did not (*n* = 619; *M* = 2.3; *SD* = 1.02). A number of 125 out of 744 (17%) consulted a medical practitioner because of PRP (Figure 1 shows the distribution of the intensity of pain between both groups). In addition, a weak but significant negative correlation between general well-being at the time of the survey and the frequency of PRP (*r* = −0.078; *p* = 0.026) was found. There was no correlation between well-being and intensity of PRP. Age and z-standardized practice time in relation to the instrument did not correlate with frequency and intensity of PRP.

In order to gain further understanding of the dependencies of PRP, we calculated multivariate regression models that considered either frequency or intensity of PRP (stepwise linear regression) as a dependent variable. The age of the respondents, their gender, their general well-being at the time of the survey, occurrence of pain independent of instrumental playing, the period during which they received lessons on their instrument, the z-standardized practice time according to instruments, the personality factors collected with the BFI-10 (Rammstedt et al., 2012), and the indication of sports as a hobby were initially selected as independent factors.

None of the regression models was able to provide a noteworthy explanation of variance. In the model predicting the intensity of PRP, only the occurrence of pain independent of instrumental playing, neuroticism, and openness loaded significantly and explained only 4 percent of variance (*F*[3,454] = 6.679, *p* < 0.001, *R*^2^ = 0.042, *R*^2^_adjusted_ = 0.036; see Table 10). In the second model predicting the frequency of PRP, this reduced to the occurrence of pain independent of instrumental playing and gender, explaining only about 5 percent of variance (*F*[2,489] = 13.714, *p* < 0.001, *R*^2^ = 0.053, *R*^2^_adjusted_ = 0.049; see Table 11). This suggests that other, individual factors such as practice habits, body posture, or vulnerability may be far more decisive for the frequency and intensity of PRP.

**TABLE 10:** Linear regression on intensity of PRP.

	***B***	**β**	***SE***	***p***
Constant	1.465			0.000
Occurrence of pain independent of instrumental playing	0.416	0.147	0.130	0.002
Neuroticism	0.117	0.104	0.052	0.025
Openness	0.135	0.096	0.065	0.039
*R*^2^	0.042			
*R*^2^_adjusted_ *F*(3; 454)	0.036			
	6.679 (*p* < 0.001)			

**TABLE 11:** Linear regression on frequency of PRP.

	***B***	**β**	***SE***	***p***
Constant	2.491			0.000
Occurrence of pain independent of instrumental playing	0.419	0.184	0.100	0.000
Gender	−0.282	−0.145	0.086	0.001

*R*^2^	0.053			
*R^2^_adjusted_ F*(2; 489)	0.049			
	13.714 (*p* < 0.001)			

*Gender reference category: male participants.*

The Kruskal–Wallis *H* test was used to identify differences between instrument groups in the frequency of PRP (Chi^2^ = 35.25; *df* = 6; *p* < 0.001). Results show that bowed string instrumentalists experience pain most frequently and differ significantly from singers (*z* = 4.484; *p* < 0.001), brass players (*z* = −3.509; *p* = 0.009), keyboard instrumentalists (*z* = 3.602; *p* = 0.007) and percussionists (*z* = −3.171; *p* = 0.032). In addition, woodwinds experience significantly more severe pain than singers (*z* = 3.423; *p* = 0.013; see Supplementary Figure 25).

Furthermore, the Kruskal–Wallis *H* test was conducted to examine the differences on intensity of PRP between different instrument groups (Chi^2^ = 23.42; *df* = 6; *p* = 0.001). Players of bowed string instruments were most affected and differed significantly from singers (*z* = 3.468; *p* = 0.011), brass (*z* = −3.098; *p* = 0.041), and woodwind players (*z* = −3.480; *p* = 0.011; see Supplementary Figure 26).

Concerning the duration of PRP, just under a third (30%) of those experiencing PRP answered “not that long” and indicated an average duration of 5.6 months (*SD* = 4.00). Just over half (52%) affirmed “already longer” and reported an average duration of 3.2 years (*SD* = 1.85). 19 percent stated that they were always in pain. All in all, approx. 70 percent indicated to have had PRP for several years or even to have always had it.

### Coping With PRP and Communication About PRP

We asked the contest’s participants what they do if they have pain while practicing. Most of them take a break (75%). Significantly fewer stated that they relax (25%) or do something else (20%). Only a small amount (14%) uttered that they practice less. A very small minority (4%) reported that they take medication to relieve the symptoms. When asked, if they have spoken to someone about PRP (multiple answers were possible), most mentioned their mother (71%), followed by their teacher (58%). Somewhat less often, they speak to their father (50%). Friends (24%) or other people are mentioned much less frequently. Younger students (9–15 years) speak more often with their mothers about pain while practicing, older students (16–24 years) with their peers. Due to multiple answers, it should be taken into account that the musicians confide in several people at the same time. For example, 64 percent of the female and 71 percent of the male participants talk to both parents about PRP. Compared with boys, girls are more likely to talk to their mother and peers about PRP. A very high percentage of participants (89%) mentioned one or more reference persons to entrust with their PRP.

An important question is to what extent the young musicians feel taken seriously when they speak about PRP to parents, teachers, or others. A little more than half of the participants (56%) feel that they are taken seriously. About one third (32%) feel that they are only partially taken seriously, and 12 percent feel they are not taken seriously. In other words, almost half of the participants in the competition feel that they are not completely taken seriously when they talk to parents, teachers, or other people about their PRP.

There is a weak but significant association between gender and whether their pain is taken seriously or not. Chi^2^-tests show that girls tend to feel taken more seriously by their reference persons (57% completely, 33% partially) with regard to their PRP than the male participants do (54% completely, 30% partially; Chi^2^ = 6.10; *p* = 0.047; Cramer’s *V* = 0.088). Teachers have a key role to play in this respect, because 94 percent of the female and 88 percent of the male participants feel taken seriously when talking to them about their PRP.

### Characteristics and Personality Traits of Participants With an Outstanding High Amount of Practice

As reported in Section “Description of the Sample, Pain Prevalence,” we see great differences or variations in weekly practice duration both between and within the different instrument groups. At the same time, the duration of the practice time is not normally distributed, but is distributed in various forms in a right-skewed manner.

In all instrument groups, some of the respondents indicated very long practice times that deviate considerably from the average value and amount up to 38:30 h per week (piano and violin). For this reason, we have identified those in the group of the competition participants who practice the most and have examined the question of whether those who practice comparatively often differ in various personality traits from those who practice less. The subgroup of those with an outstanding high amount of practice was formed by musicians who practice more than 21 h per week. This corresponds to about twice the average value of the instrument-specific practice time for instruments that require intensive practice such as piano (*M* = 10:50, *SD* = 7:48) or violin (*M* = 9:57; *SD* = 6:45; see Table 3) and about four times the average value for instruments that are usually practiced for less time such as the flute (*M* = 4:46; *SD* = 4:28).

Practicing 21 h per week means spending more than 3 h each day. This is remarkable for young people who attend secondary school, often having lessons in the afternoon, most of them doing sports (73% of those who practice a lot) and perhaps even meet friends. A total of 33 participants were identified as a subgroup of those with an outstanding high amount of practice. Their ages range from 13 to 21 years, the average age is 17 years (*SD* = 1.82). Two thirds are female, one third male. Most of them (*n* = 15; 45%) play the piano, followed by the violin (*n* = 7; 21%). Significantly fewer of those playing cello and harp (*n* = 3 for each), trumpet and horn (*n* = 2 for each), and flute (*n* = 1) are found among them. The uneven distribution is on one hand due to the fact that piano, flute, and violin are the most frequently represented instruments in the overall sample (see Table 3), and on the other hand due to the fact that piano and violin are among the instruments on which, for physiological reasons, it generally takes the longest to practice. A good half (52%) takes part in the solo contests of the competition, the other half (49%) in the ensemble contests.

**FIGURE 2:**
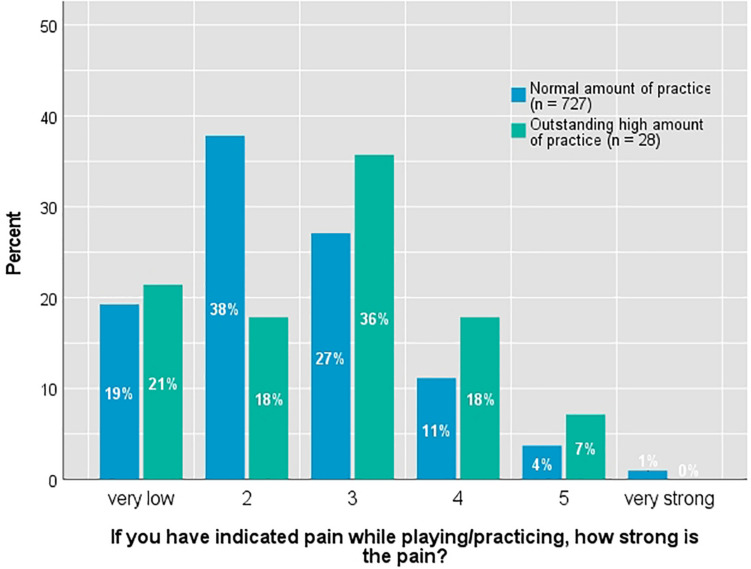
Distribution of pain intensity in differentiation according to amount of practice.

By far the largest part (94%) of this sub-sample indicates already having pain when practicing or playing the instrument. This is significantly more than the average of the overall sample across all instruments (76%; *N* = 1.143). The indicated intensity of pain is *M* = 2.7 (*SD* = 1.21; *Mdn* = 3.0) on the six-level scale (the distribution is shown in Figure 2). Half of this sub-sample (50%) stated having endured the pain for a longer time, 13 percent say they have always had the pain. Nearly one-third (32%; *n* = 10) have already consulted a physician as a consequence. Some also specified they have pain that has nothing to do with practice (10%; *n* = 3). In individual cases (13%; *n* = 4), pain medication is also taken, although it is not clear how often the medication is due to PRP or other pain.

The most frequently mentioned pain areas (defined as the sum of the mentions in the categories frequent/often/always) are neck and shoulder area (70%; all participants with PRP: 69%), back (60%; all participants with PRP: 55%), arms (42%; all participants with PRP: 54%), wrist (32%; all participants with PRP: 56%), hands (30%; all participants with PRP: 48%), and fingers (25%; all participants with PRP: 51%). The majority of these participants (71%) stated that they took a break from PRP as a coping strategy. Other coping strategies such as the Alexander Technique, movement and walking, or other relaxation exercises are cited by 42 percent of those who practice comparatively often. Just under 10 percent declared they practice less.

In addition to the aspects described above, it was examined whether those with an outstandingly high amount of practice differ from the other participants with respect to the personality dimensions openness, conscientiousness, extraversion, agreeableness, and neuroticism (Big Five personality model). For this purpose, the scores from the Big Five Inventory-10 (Rammstedt et al., 2012) were used, which were tested as dependent variables for group differences (*t*-test). The sub-sample of those with an outstanding high amount of practice differs from the rest of the sample by significantly higher values in the dimensions extraversion (*t* = −2.07; *df* = 1,023; *p* = 0.039; *d* = −0.379), conscientiousness (*t* = −2.44; *df* = 1,040; *p* = 0.015; *d* = −0.417), and openness (*t* = 2.76; *df* = 1,038; *p* = 0.006; *d* = −0.499).

All in all, these findings show that a very high number of practice hours is associated with a noticeably increased risk of PRP. A comparison on the level of individual instruments is not possible due to the too small number of cases in the group of those who practice comparatively very often.

The intensity of pain in this sub-sample (*M* = 2.7; *SD* = 1.21) does not differ significantly from the group of those with a generally high amount of practice overall (*M* = 2.6; *SD* = 1.15), which, however, is significantly higher (*p* = 0.004) than that of the overall sample of participants in the competition (*M* = 2.4; *SD* = 1.06).

With regard to communication about PRP, the mother is more often named as the discussion partner (79%) than the father (49%). In 64 percent of cases, the instrumental teacher is spoken to, in 30 percent of cases a friend is spoken to. The vast majority of them believe that the pain when practicing is taken seriously (61%). A third (36%) feels that it is only partially taken seriously. The differences to the overall sample (see above) are only relatively small.

## Discussion

The results of the present study show that a total of 76 percent of a large sample of high-performing young musicians reports experience with PRP (see section “Description of the Sample, Pain Prevalence”). The average prevalence rises with age from 71 percent (9–13 years) to 85 percent (18–24 years). Apart from age, the prevalence of PRP we observed varies between 43 and 100 percent depending on the instrument and duration of practice (see section “Instruments, Time of Weekly Practice, and PRP”). These results are consistent with findings by Kaczmarek (2012) and Nawrocka et al. (2014). They demonstrated that high-performing young musicians who are used to practice a lot, are more likely to report PRP than children and adolescents who play an instrument but are not high-performing musicians. With regard to instrumental teaching, it is noteworthy that a relatively high prevalence of PRP of 71% can already be observed in the youngest group of 9–13 years. This means that mindfulness of PRP cannot begin early enough. Especially young musicians, who play instruments with a relatively high risk of PRP (e.g., high strings, oboe, clarinet, harp, and accordion) should be monitored with regard to the occurrence of PRP, in order to take countermeasures if necessary.

We used multiple regression models to examine possible correlations between frequency and intensity of PRP as dependent variables on one hand, and variables such as age, gender, general well-being at the time of the survey, occurrence of pain independent of instrumental playing, the time period during which they received instruction on their instrument, the z-standardized exercise time according to instruments, personality factors, and sports activities on the other hand, but we yielded only weak results and revealed hardly any significant findings (see section “Localization, Frequency, Intensity, and Duration of PRP”). Even when existing pain, which was independent of music-making, was included in the regression models, no really substantial correlations could be found. This could indicate that the prevalence of PRP and the intensity of PRP are based on a very individual interaction of different factors whose patterns are difficult to predict.

Being able to play an instrument at this high level requires an above-average amount of practice already in childhood and youth. The level of exercise with which high performance is achieved can vary widely. When looking at the ranges, mean values, and standard deviations of the practice time in this study, the following is noticeable: Firstly, the mean values of the practice duration vary considerably between different instruments. Second, the variance in practice duration is very large within the different instruments (see Table 3; the individual distribution of weekly practice time for all instruments can be found in the Supplementary Material). Some players with the same instrument practice very little, others show comparatively high practicing efforts. Therefore, it is necessary to distinguish (not only with regard to the prevalence of PRP) between two different types or aspects of the variance in duration of practice: (a) the variance in instrument-related duration of practice between different (types of) instruments (e.g., keys, strings, wind instruments, voice, etc.) and (b) the variance of individual practice time within the same instrument or type of instrument, which is mainly due to individual factors such as practice habits, endurance, motivation, and actual physical-psychological constitution, etc. These two types of variance in practice time are mixed and cannot be clearly separated.

Concerning the possible influence of personality traits, the sub-sample that practices the most differs from the rest of the sample by significantly higher values in the dimensions extraversion, conscientiousness, and openness (see section “Characteristics and Personality Traits of Participants With an Outstanding High Amount of Practice”). It is not easy to explain this finding, since studies on personality traits of musicians usually refer to (adult) musicians compared to non-musicians and less to children and young people (e.g., Hargreaves and Lamont, 2017). Perhaps the high amount of time spent practicing is an expression of conscientiousness and perfectionism. A high degree of extroversion could also be an expression of the need to present oneself on stage in order to experience and perform power (cf. Bullerjahn et al., 2020). The performance on stage depends on conscientious preparation, which requires a high degree of practice. Openness, on the other hand, together with extroversion may be an advantage for an optimal stage presentation. It is an interesting question whether those who practice the most and show a stronger emphasis on the dimensions of extraversion, conscientiousness and openness will have more success in competition than other participants. For reasons of anonymity it was not possible to collect and include the results of the competition in this study. To what extent these speculations are correct, future studies would be necessary to examine.

In the studies by Kaczmarek (2012) and Nawrocka et al. (2014), the subjects are partly pre-professional musicians who differ from other young musicians in their high level of performance. At the same time, the high-performing young musicians are not part of a homogeneous group concerning their practice time, since the average values of the practice time reported by them are quite different. Unfortunately, the practice time in existing studies is collected or reported in different ways: average number of minutes per day, number of hours per day, or number of hours per week. To be able to compare the different values from these studies, we calculated the practice time per week on the basis of the reported practice time per day and the specified number of practice days per week. In the study by Nawrocka et al. (2014), the time spent practicing is reported in minutes per day (p. 66). Since the subjects stated that they would practice most days of the week (p. 65), we have assumed 6 days of practice, which are also reported in the other studies (see above). According to this, Nawrocka et al. (2014) report 9:25 h/week. Our study found averages of 7:18 h/week for all instruments.

Substantially higher practice time is found by Kaczmarek (2012; average practice time almost 20 h/week) and Heye (2019, p. 235; average practice time 24 h/week) in comparable samples of highly talented young students studying at a music academy. This high weekly amount of time spent practicing is comparable to the practice time of students at music universities who are pursuing a professional career as musicians (cf. Jørgensen, 1997, p. 132; Macnamara and Maitra, 2019, p. 12). Such high and even higher duration of practice time is also found in some cases in our study (see Table 3).

Overall, these data draw a somewhat complicated picture. On one hand, our data show a clear relationship between the duration of practice and prevalence of PRP (see Table 4) and confirm results from Robitaille et al. (2018) as well as from Ioannou et al. (2018). On the other hand, the prevalence of pain in the study by Kaczmarek (2012) should be significantly higher than the very high prevalence of PRP in the study by Nawrocka et al. (2014), due to the differences in the practice times.

An obvious conclusion is that there is no simple linear relationship between the duration of exercise and the prevalence of PRP. This is also supported by an observation from Nawrocka et al. (2014), who reported that the male young musicians practiced more but had a lower prevalence of PRP (see p. 68). Therefore, in the context of research into prevalence of PRP, it is necessary to consider not only the instrument and duration of practice as a factor, but also qualitative aspects such as practice habits or practice strategies, attention to breaks, warm-up/cool-down, mental practice, posture, etc. (e.g., Kaczmarek, 2012; Ioannou and Altenmüller, 2015; Ling et al., 2018).

Nawrocka et al. (2014) point out:

“[…] in case of young instrumentalists, it is important to keep in mind that they can make some technical errors, while in training, especially during their home practice, due to lack of proper supervision. This is considered to pose a particular risk for the musculoskeletal system. These errors can also be associated with some difficulties with adjustment of an instrument (its size, weight, etc.) to some parameters of the children’s somatic frame. These mistakes are almost unavoidable at certain stages of musical education.” (Nawrocka et al., 2014, p. 68).

It is difficult to decide whether such technical errors are really unavoidable or would be less significant as triggers of PRP, if the practice were more strictly supervised by teachers. Fry (1987) remarks on the role of technical errors in the development of PRP: “The student’s technique is often overdrawn as being the only factor that matters” (Fry, 1987, p. 39). In addition to this technical factor, two other factors in his study with Australian instrumental students appeared to be decisive. Obviously, some students are more vulnerable to developing PRP than others. Fry considers this a genetic factor which cannot be influenced. Furthermore, Fry (1987) found a direct correlation between the combination of increased duration and intensity of practice on one hand and the occurrence of complaints on the other. Therefore, he states: “Intensity × time of practice is a totally controllable factor and seems the most important of the three factors.” (Fry, 1987, p. 39). All in all, our findings clearly show that the prevalence of PRP should be considered in a more differentiated way for each instrument and its specific requirements, resulting in different amounts of time spent practicing.

With regard to the relationship between practice and the occurrence of PRP, it is common and obvious to primarily consider the duration of practice and the type of instrument. In addition, however, the quality and method of practicing also play a decisive role in the effectiveness of practicing. This includes practice strategies such as repetitive, non-varied practice vs. mental training strategies, intentions, behaviors, and emotions, and as well the subdivisions in practice time or the complexity of the musical pieces. For instance, it appears that the use of mental strategies in practice is a more important predictor of the success of practice than the frequency of practice (McPherson, 2005). A more recent study (Mornell et al., 2020) with elite performers (average age: *M* = 26.9 years, *SD* = 7.41) showed positive correlations between the effectiveness of practice, the degree for progress and the use of mindful deliberate/intentional practice.

These and other studies underline the important role of the quality of practice (see, e.g., Jørgensen and Hallam, 2009) and show that the quantity of practice is not significantly related to the quality of performance (Williamon and Valentine, 2000). To our knowledge, however, there is no study that has investigated possible correlations between the quality of practice or different practice strategies on the one hand and PRP on the other hand, although it can also be assumed that interindividual differences and possible interactions with other variables (e.g., kind of instrument) may play an important role.

Certainly it is important that children and young instrumentalists with PRP speak to their parents and teachers about their complaints, especially when the pain is more pronounced. The data of the present study show that the majority of the young musicians concerned talk with their parents and teachers about their PRP (see section “Coping With PRP and Communication About PRP”). But we have no information about the way PRP is talked about, how parents and teachers react to it. However, it is a problem that 32 percent felt that their complaints were not completely taken seriously, and 12 percent did not feel taken seriously at all. This corresponds to the observations Gembris and Ebinger (2017) had already made in their study with non-expert instrumental students: on one hand, a good half (56%) felt that their complaints about PRP were taken seriously. On the other hand, 31 percent felt that their complaints were only partially taken seriously, and 19 percent felt that they were not taken seriously at all (Gembris and Ebinger, 2017, p. 143).

Instrumental students may also be afraid to speak about their PRP. In particular, cynicism, the accusation of hysteria, or misbehavior toward instrumental students can have a negative impact on the further development of PRP, as Fry (1987) points out. The fear of losing the teacher’s goodwill may cause students to continue practicing and the symptoms of PRP may become seriously worse (cf. Fry, 1987, p. 38). This can also lead to psychological problems such as loss of self-esteem (p. 40). Apparently, there is a problem of communication between those young musicians experiencing PRP and relevant authority figures which has not yet been adequately considered in research or musical practice. An improvement in the communication about PRP between instrumental students, teachers, and parents could – through appropriate treatment, countermeasures, and prevention – contribute to a reduction of the high prevalence rates of PRP already among young musicians. This is also of long-term importance, especially for those young musicians who are pursuing a career as professionals.

Another aspect may be interesting. Considering the prevalence of PRP, it is important to take into account that a significant proportion of children and young people complain about pain that is independent from making music. In our study, 22% of respondents said they had pain that was independent of making music (see section “Description of the Sample, Pain Prevalence”). A review by King et al. (2011) demonstrates that the prevalence of headache, abdominal pain, and back pain in children and adolescents varies widely in international research (headache: 8–83%; abdominal pain: 4–53%; back pain: 14–24%; musculoskeletal pain: 4–40%; multiple pains: 4–49%; other pains: 5–88%). The reasons for this considerable diversity in the results may be different data sources, age groups, time of measurement, and operationalization of pain (see also Aartun et al., 2014).

Aartun et al. (2014) reported in a Danish longitudinal study that about 9 out of 10 school children aged 11–15 years have experience with neck and back pain (neck pain, low back pain, and mid back pain). For most of these complaints, however, they are mild, relatively rare, and of low intensity. Neck pain was most common, followed by mid-back pain and low back pain. (This corresponds to our findings concerning PRP).

A comprehensive longitudinal epidemiological study in Germany (KiGGS Health Survey; see Krause et al., 2017) with almost 12,368 children (0–17 years) showed that a total of 18 percent of the 11–17-years-olds had repeatedly experienced back pain in the past 3 months. Girls (22%) showed a significantly higher prevalence than boys (14%). A problematic aspect of recurrent pain is that it is associated with loss of quality of life, anxiety, and depressive symptoms compared to peers without recurrent pain (p. 424). The question arises whether this could possibly also apply to PRP. In addition, pain may persist from a young age into adulthood (p. 416; see also Aartun et al., 2014). In general, the prevalence of pain in children and adolescents increases with age. This also raises the question of possible parallels with PRP.

A recent study (Joergensen et al., 2019) on spinal pain (low back pain and middle back pain) in children within the Danish National Birth Cohort with 46,726 adolescents aged 11–14 years concluded that almost 10 percent of the boys and 14 percent of the girls reported severe pain. Again, an increase with age was observed. “In addition, children in more disadvantaged families were more likely to experience spinal pain” (p. 704; see also Ballenberger et al., 2018). Batley et al. (2019) were recently able to show that other social factors such as increased loneliness and lower acceptance by other students as well as psychological factors such as increased nervousness, more frequent low/bad mood, and difficulty sleeping are associated with spinal pain in adolescents (average age *M* = 12.6 years, *SD* = 0.61). These findings raise the question of the possible role of psychological and social factors in the genesis of PRP (see Ballenberger et al., 2018).

## Limitations

When considering the results, some limitations of the study should be taken into account. We conducted the present study of PRP in the context of an extensive study of highly gifted young musicians, in which PRP is only one of many topics. Therefore, there was only limited space available for questions on PRP within a very long, 17-page questionnaire. For this reason additional special questions, e.g., on the quality of practice could not be included. Possibly, additional explorative case studies would offer suitable approaches for tracking down individual patterns and gaining better understanding of the relationships between exercise behavior and PRP. Furthermore, investigating the relationships and parallels between PRP and non-musical pain in children and adolescents exceeded the possibilities and limits of this study. This requires further research by pain experts.

The questionnaire is standardized but is not yet a validated instrument compared to other pain scales. One reason why we did not use existing validated questionnaires for the assessment of PRP is that the existing instruments are designed for adults and seemed less suitable for our context with children and adolescents. A second reason why we adopted the questions from the study by Gembris and Ebinger (2017) is that we were interested in having the possibility of a direct comparison of the results of both studies. A third, more pragmatic reason is the length of the questionnaire in which the questions on PRP had to be embedded. Finally, due to various circumstances, the questionnaire had to be created in a very short time, so that there was no possibility for validation.

The sample includes players of different musical instruments from a variety of instrument groups. However, the different instruments are represented in the sample in very different proportions, ranging from *n* = 14 (guitar) to *n* = 208 (piano). The selection of the different instruments in the sample could not be done systematically. The different case numbers for the various instruments are due to the fact that the 2017 “Jugend musiziert” contest was not meant for all instruments, but for specific instruments in both the solo and ensemble categories. A very different number of young musicians participated in each of the contest categories (for details see Gembris and Bullerjahn, 2019; Bullerjahn et al., 2020).

Because of the large disparity between number of players for the different types of instruments, the data and results of analysis for the different instrument groups may be generalized to varying degrees. Due to the relatively small number of cases in some instrument groups (e.g., guitar, trombone, and oboe), the possibilities of statistical analysis are limited to some extent with regard to the subdivision into subgroups (e.g., age, gender, high amount of practice vs. low amount of practice, etc.) and the associated study of possible influencing factors and comparisons. In general, correctly recording the duration of practice is a problem, which may be due to different definitions of practice (e.g., Macnamara and Maitra, 2019), estimation errors, incorrect answers knowingly or unknowingly given, etc.

Furthermore, the sample consists of particularly talented, high-performing young musicians who show a musical commitment far above average. While the results should be highly informative for this group, they cannot readily be transferred to non-expert music students.

## Conclusion

Three-fourths of the young, high-performing musicians we surveyed indicate experience with PRP. This fact is in line with other studies (e.g., Kaczmarek, 2012) and should give reason to pay more attention to this topic in instrumental instruction. We suggest that the topic of musicians’ health and prevention should already be included in lessons with young instrumentalists. A high number of practice hours is associated with a noticeably increased risk of PRP. Regarding localization and frequency of PRP, our results are in line with many other studies indicating that musculoskeletal problems are the most common physical complaints of performing musicians.

Most of the young musicians experienced mild (57%) or moderate (38%) intensities of PRP (see also Table 9 and Supplementary Figure 26). Therefore, PRP should be considered in a differentiated way. Only a very small part of 5 percent in our study had severe pain.

In most cases, PRP will presumably not cause severe suffering. Many music school students report that PRP disappears after playing or does not last very long after playing (Gembris and Ebinger, 2017). A crucial question is to what extent the quality of life and making music is affected by intensity, duration, and frequency of PRP. A small part of 5 percent among young high-performing musicians in our study reports high intensity of PRP, 70 percent have been experiencing PRP for years. It is important to identify especially those young musicians who suffer severely and have had complaints over a longer period of time, in order to treat the complaints and counteract it by changes in practice.

Since PRP is obviously not only widespread among professional musicians but also among young instrumentalists, it is generally necessary to counteract it by providing information, by teaching adequate practicing techniques, by scheduling a warm-up, a cool-down, and breaks, correcting incorrect postures, self-observation while practicing, etc. Therefore, information about musician health and prevention should already be included in lessons with young instrumentalists.

The results of the present study confirm the need to improve communication on PRP between students, teachers, and parents (see also Gembris and Ebinger, 2017). Not all young musicians who report complaints about PRP talk about it with their parents, teachers or other people. If they talk to teachers or parents about PRP, a large portion (44%) feel that they are not really taken seriously. In general, it is problematic when children or young people feel that they are not being taken seriously. In our context, it seems to be important in terms of prevention and coping to ensure that parents and teachers take their children and students seriously about PRP. This can probably best be achieved by integrating the topic of PRP and healthy music-making into the training of future instrumental teachers and offering them appropriate continuing education courses.

We have some doubts regarding the opinion of parents who claim that PRP is an inevitable problem which must be endured by musicians (Ackermann and Driscoll, 2013). It may be that complaints with PRP cannot always be avoided, especially during intensive musical training. However, they should not simply be accepted, but should be used as an opportunity to change habits in such a way that PRP is avoided or reduced.

Since a retrospective survey on former “Jugend musiziert” participants indicates that approx. 50 percent of them will become professional musicians (Gembris et al., 2019), it is especially important to recognize PRP in the case of these high-performing adolescents, who are more frequently affected by PRP than other music school students. We have empirical evidence, that for orchestral musicians suffering from PRP, the history of PRP goes back to their youth (Gembris et al., 2018). If we assume that PRP may be similar to non-musical pain in childhood and adolescence that tends to persist and increase into adulthood (see Aartun et al., 2014; Krause et al., 2017), it is important to counteract PRP as early as possible. However, this speculation requires further empirical verification in future studies. Another important task for future research is to investigate communication about PRP more thoroughly. As the study by Ackermann and Driscoll (2013) indicates, parental education and own musical experiences play an important role in the appraisal of PRP, which is still largely unexplained.

We do not really know how many young musicians have problems with PRP but do not talk about it. If there were more information about PRP, if PRP were not perceived as a flaw (cf. Fry, 1987) and if talking about it were more common, then these young musicians could be helped better. As other authors have suggested (e.g., Fry, 1987; Spahn, 2011), appropriate prevention and information on how to avoid PRP and, where appropriate, offering suitable treatment options should be part of good instrumental teaching.

## Data Availability Statement

The datasets for this manuscript are not publicly available because the participants have been assured that the data will not be passed on to third parties and because the data is property of the University of Paderborn. Requests to access the datasets should be directed to the corresponding author.

## Ethics Statement

The study was conducted in full accordance with the Ethical Guidelines of the German Association of Psychologists (DGPs) and the German Association of Psychologists (BDP) as well as the Ethical Principles of Psychologists and Code of Conduct of the American Psychological Association (APA). These guidelines suggest that for the type of research reported here, a formal ethics approval is not necessary. This is due to the fact that the study only made use of completely anonymous questionnaires and thus, no identifying information was obtained from the participants. Moreover, participants and their parents were informed about the aim of the questionnaire, the anonymity of the data, and that participation was voluntary. In accordance with the ethical principles mentioned above, it was not required to obtain written informed consent.

## Author Contributions

HG, CB, JM, and AH conceived and designed the questionnaire and performed the survey. HG, JM, and AH analyzed the data and wrote the manuscript. All authors contributed to the article and approved the submitted version.

## Conflict of Interest

The authors declare that the research was conducted in the absence of any commercial or financial relationships that could be construed as a potential conflict of interest.

## Footnotes

1 The format 6:18 is used here and elsewhere when hours:minutes are meant. The practice time reported here differs from that reported by Bullerjahn et al. (2020) for the superordinate large-scale study. This is due to the fact that, for methodological reasons, the calculation in the present paper only includes respondents who stated that they practiced at least half an hour per week, as well as instruments represented in the data set with a minimum number of *n* = 10.
