# Supplementary material for: High-Performing Young Musicians’ Playing-Related Pain. Results of a Large-Scale Study
Source: Front Psychol. 2020 Dec 16;11:564736. doi: 10.3389/fpsyg.2020.564736 (PMC7772153; doi:10.3389/fpsyg.2020.564736)
Supplement: Supplementary file 1 [file Data_Sheet_1.pdf]

Supplementary Material

1 Supplementary Figures

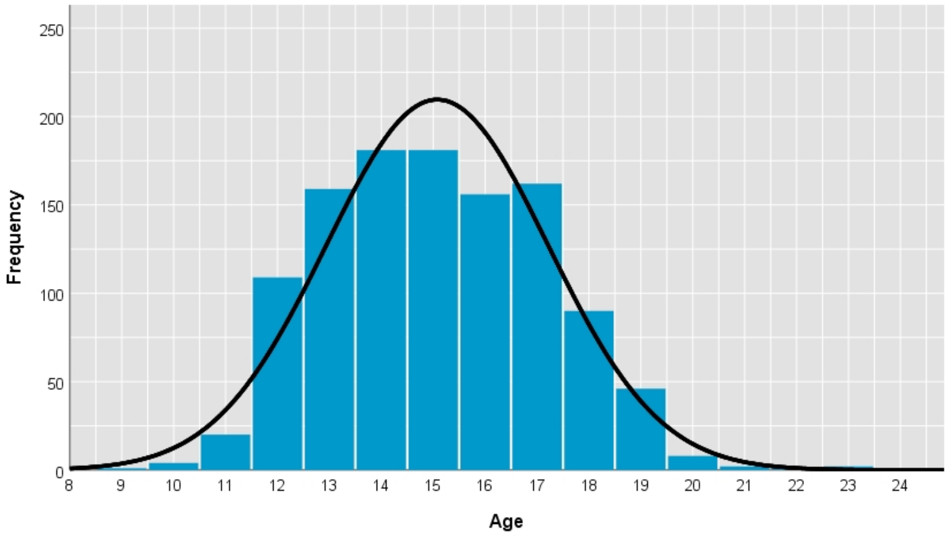

Supplementary Figure 1. Age distribution (*N* = 1,143).

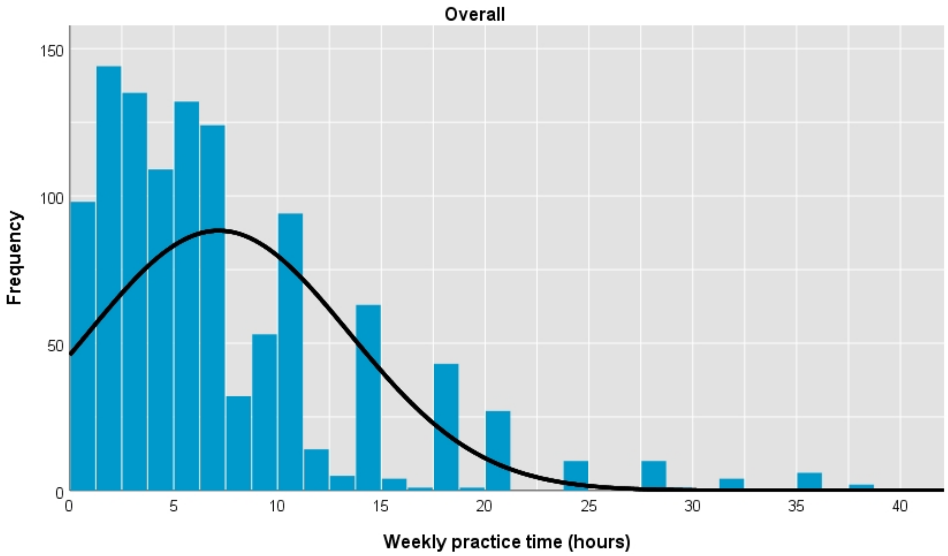

Supplementary Figure 2. Distribution of the weekly practice time, full sample (*n* = 1,070).

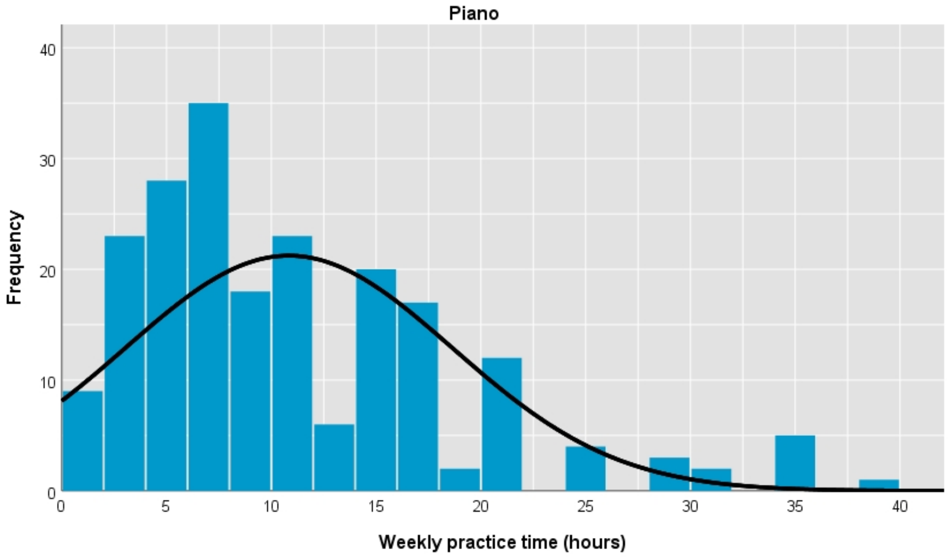

Supplementary Figure 3. Distribution of the weekly practice time, piano (n = 208).

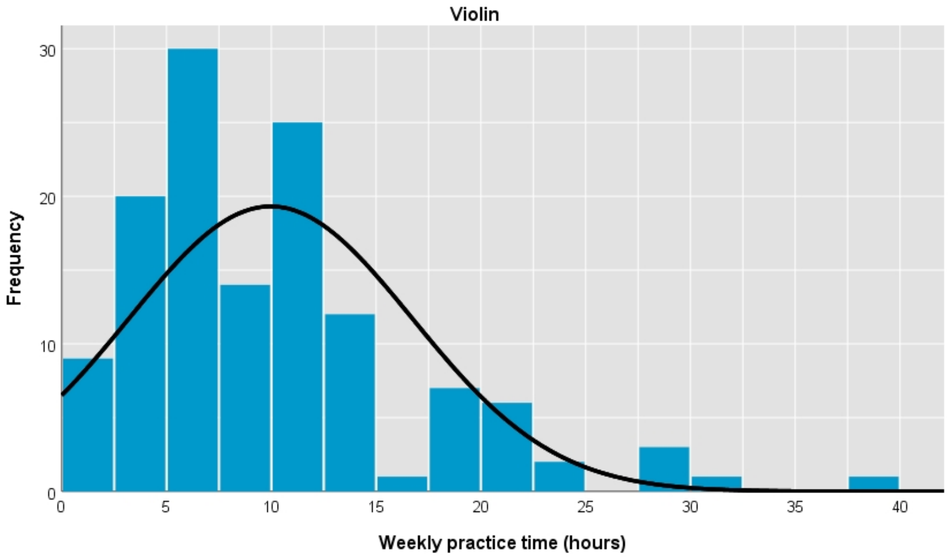

Supplementary Figure 4. Distribution of the weekly practice time, violin (n = 131).

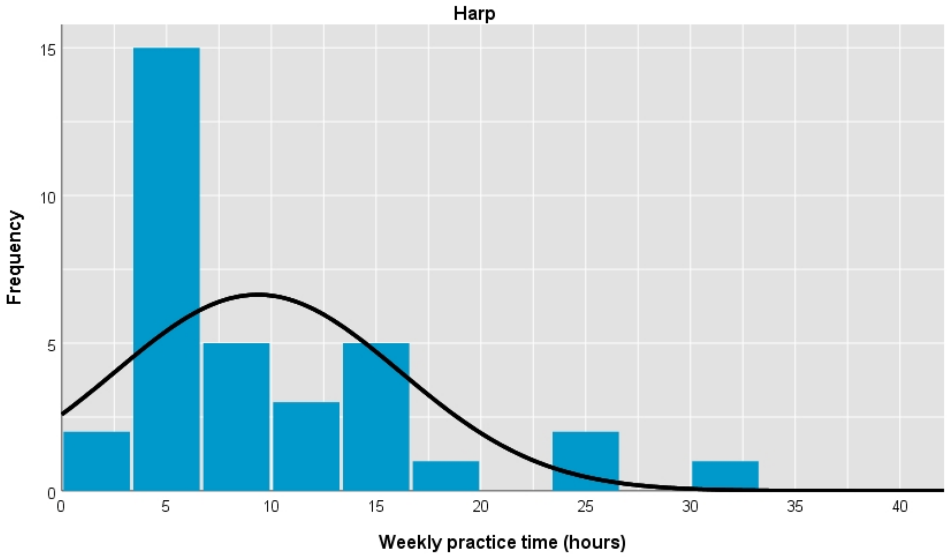

Supplementary Figure 5. Distribution of the weekly practice time, harp (n = 34).

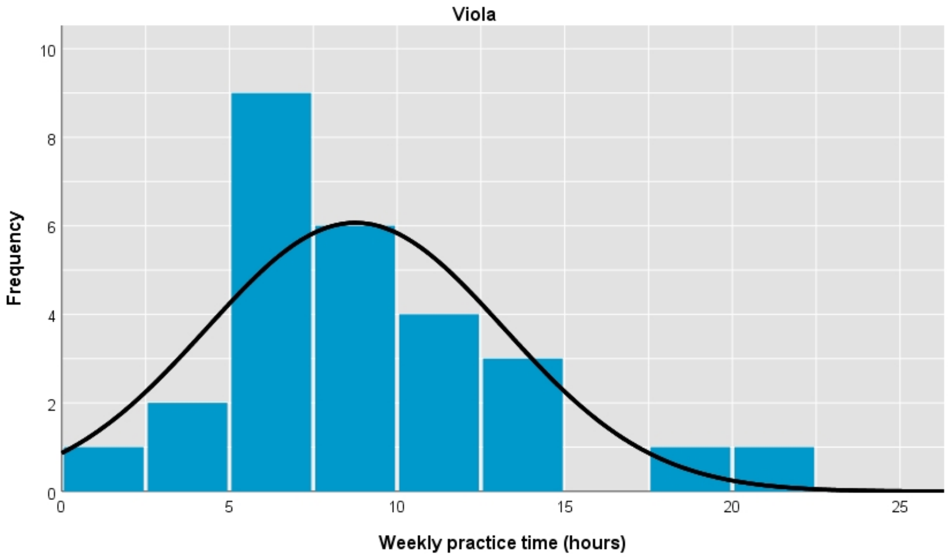

Supplementary Figure 6. Distribution of the weekly practice time, viola (n = 27).

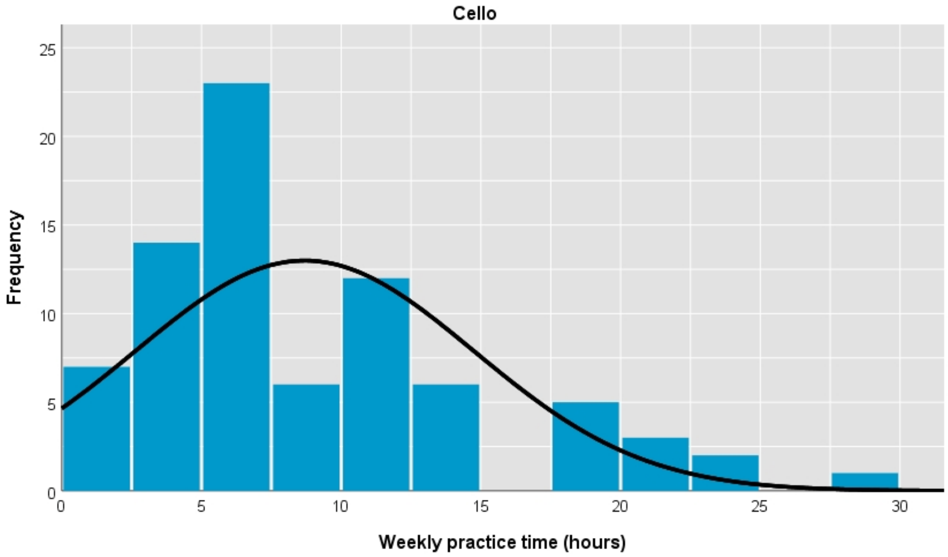

Supplementary Figure 7. Distribution of the weekly practice time, cello (n = 79).

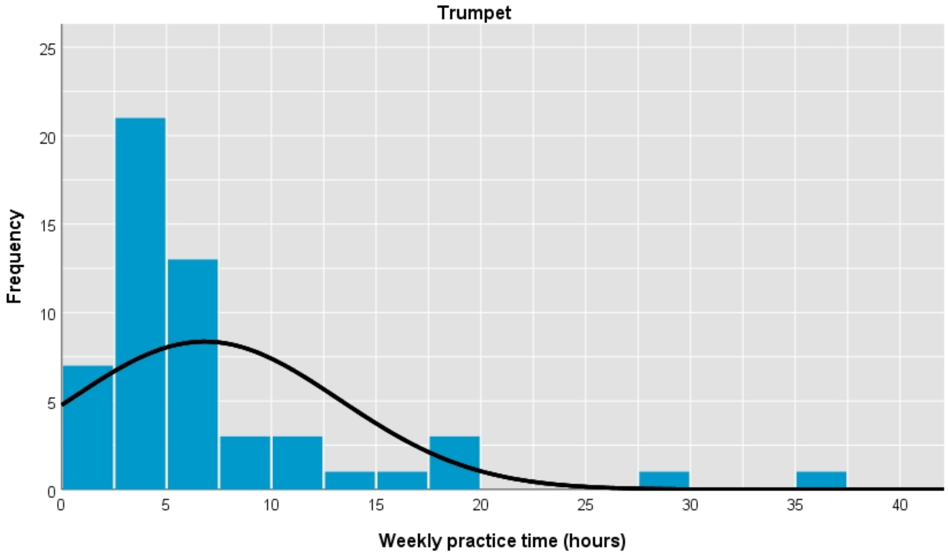

Supplementary Figure 8. Distribution of the weekly practice time, trumpet (n = 54).

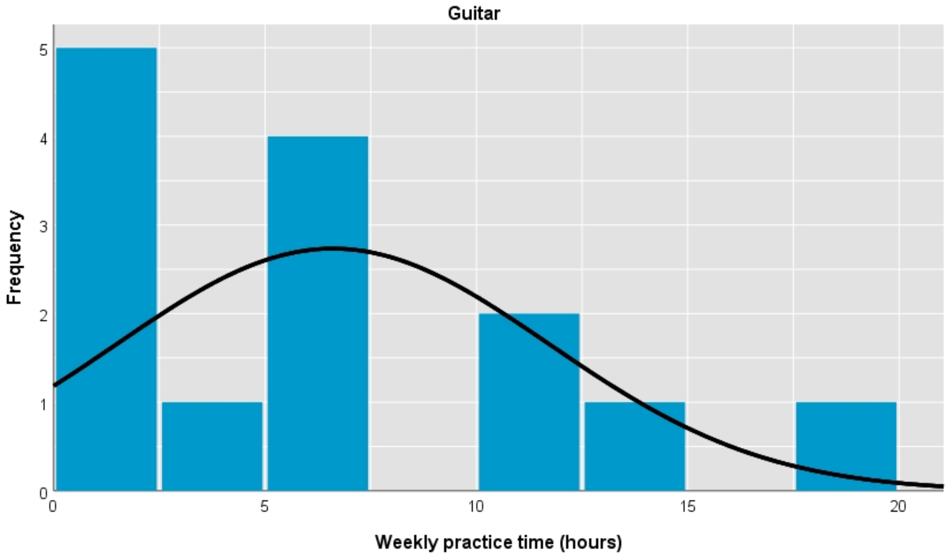

Supplementary Figure 9. Distribution of the weekly practice time, guitar (n = 14).

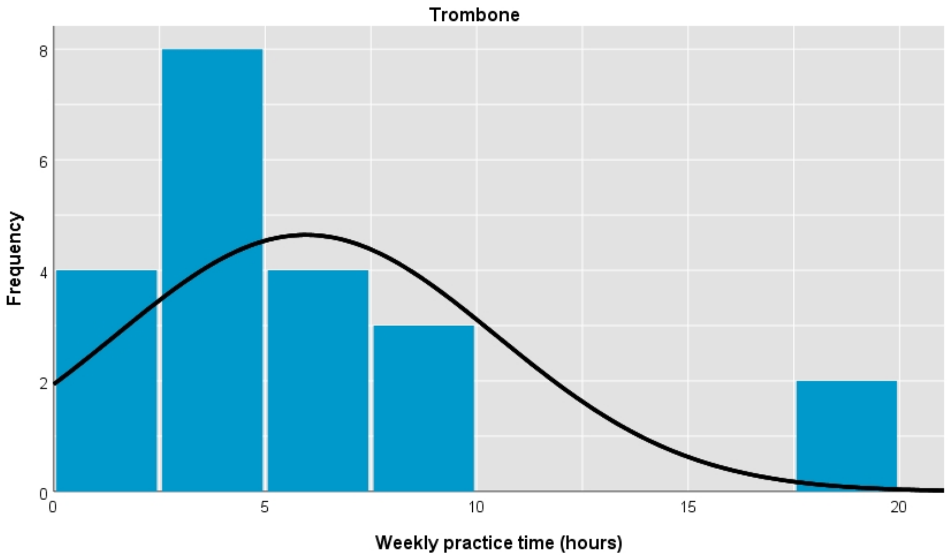

Supplementary Figure 10. Distribution of the weekly practice time, trombone (n = 21).

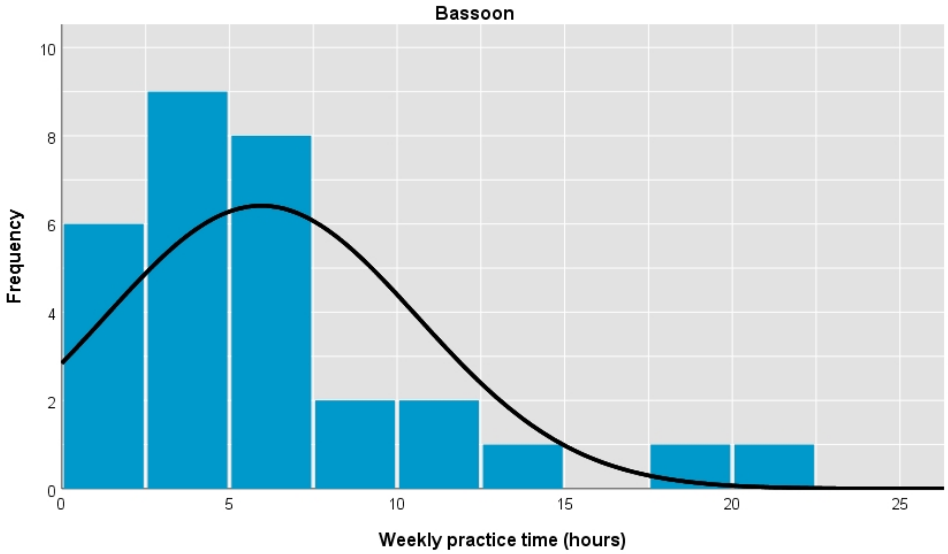

Supplementary Figure 11. Distribution of the weekly practice time, bassoon (*n* = 30).

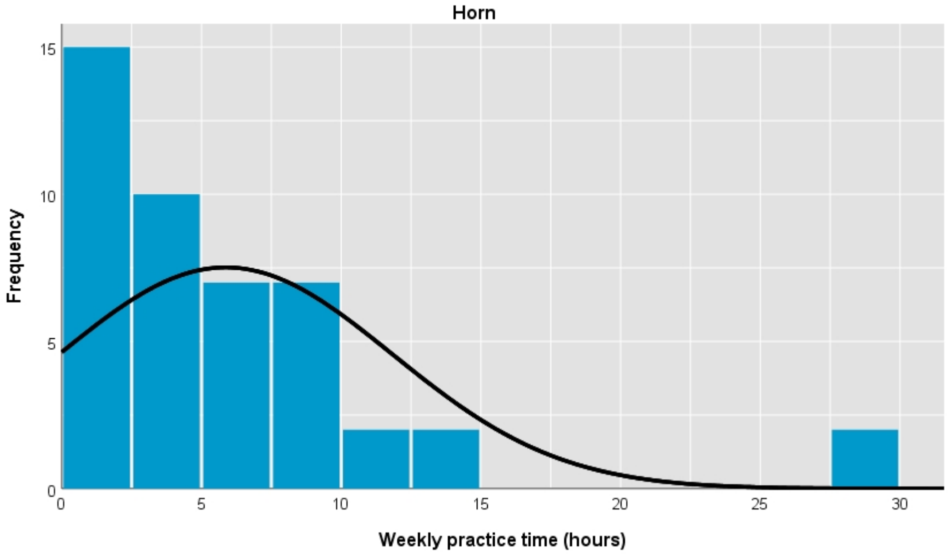

Supplementary Figure 12. Distribution of the weekly practice time, horn (*n* = 45).

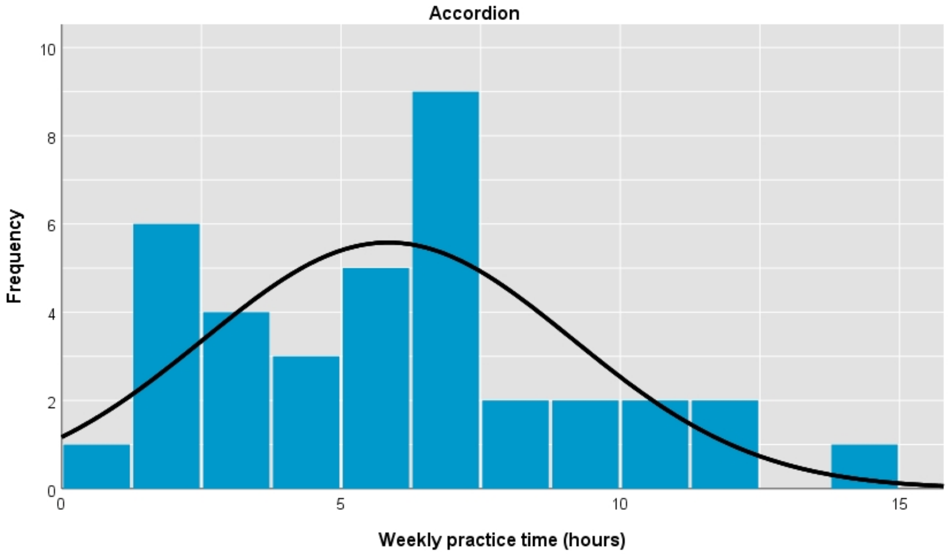

Supplementary Figure 13. Distribution of the weekly practice time, accordion (*n* = 37).

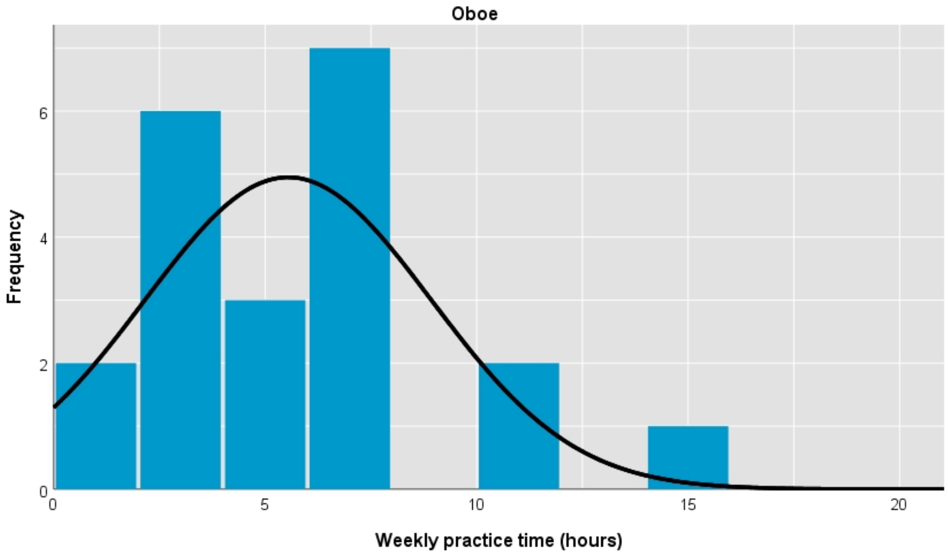

Supplementary Figure 14. Distribution of the weekly practice time, oboe (*n* = 21).

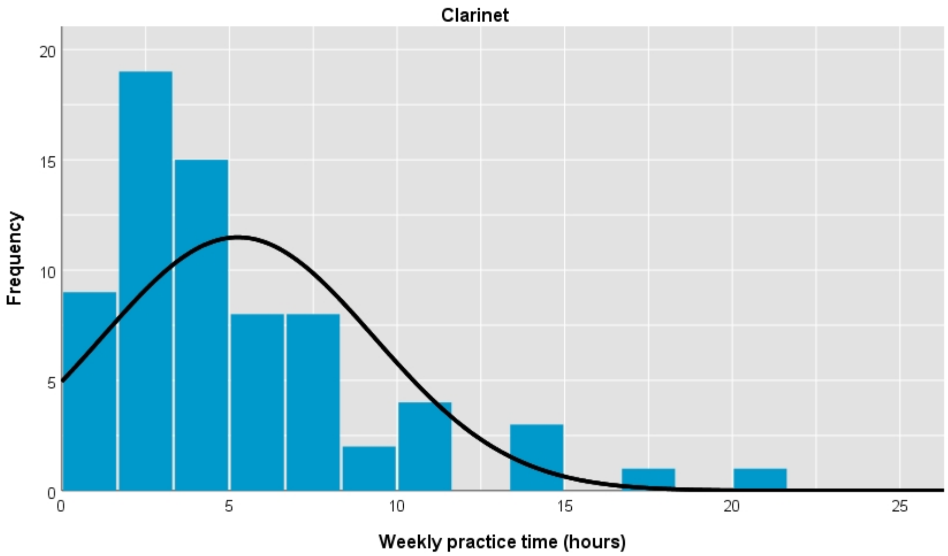

Supplementary Figure 15. Distribution of the weekly practice time, clarinet (n = 70).

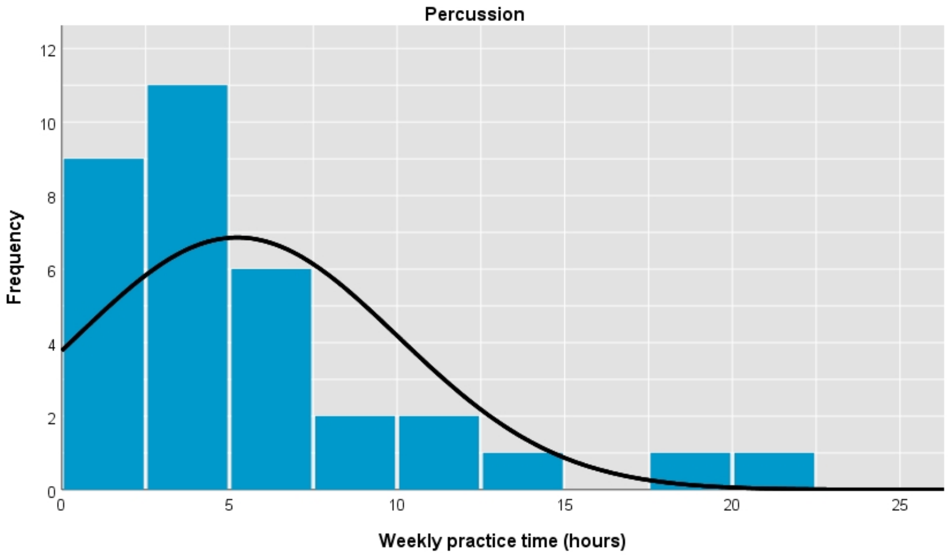

Supplementary Figure 16. Distribution of the weekly practice time, percussion (n = 33).

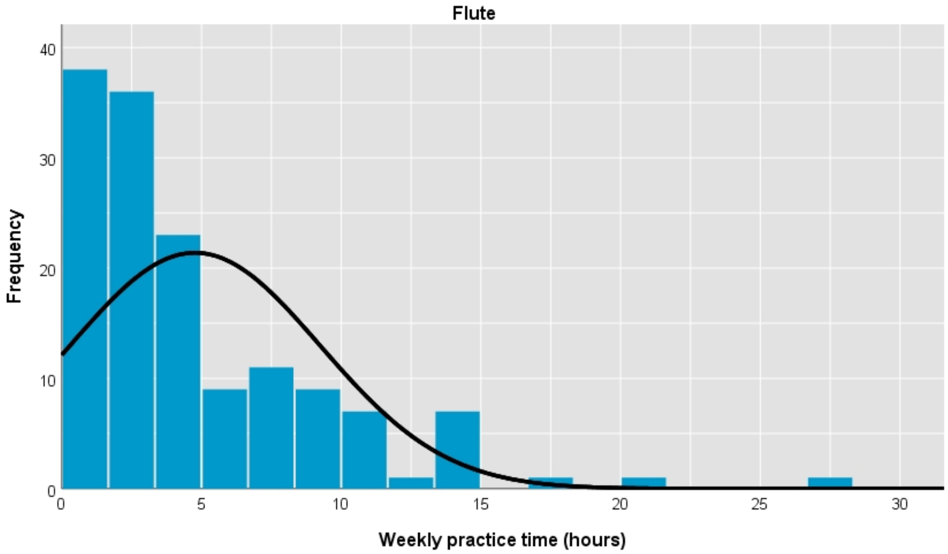

Supplementary Figure 17. Distribution of the weekly practice time, flute (*n* = 144).

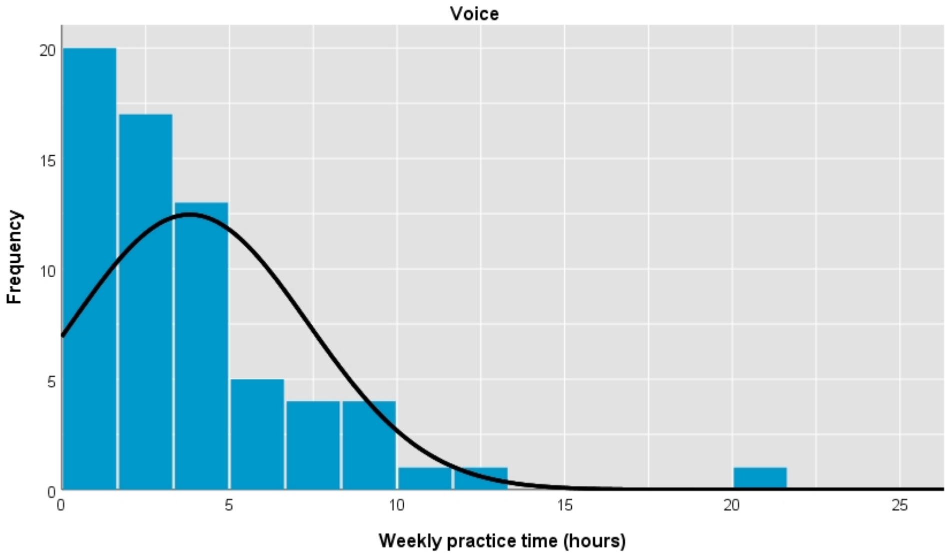

Supplementary Figure 18. Distribution of the weekly practice time, voice (*n* = 66).

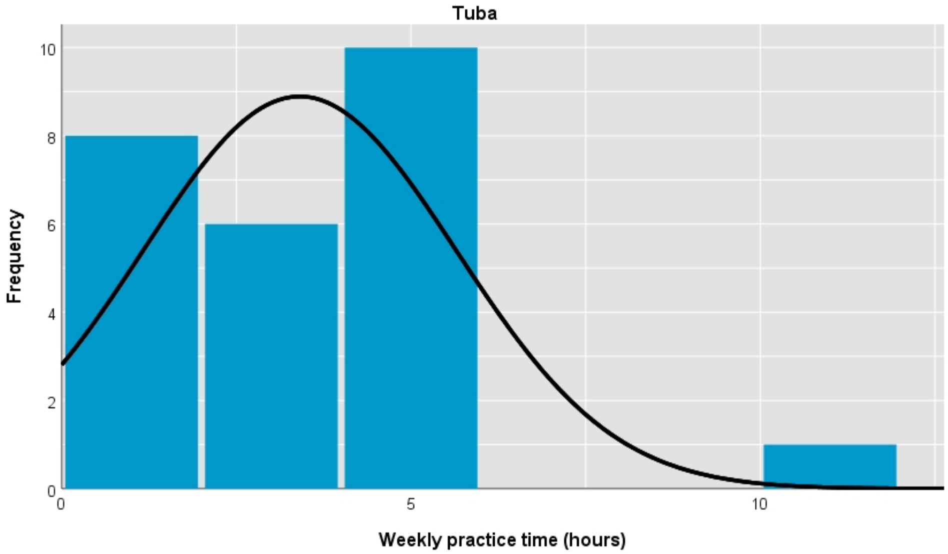

Supplementary Figure 19. Distribution of the weekly practice time, tuba (n = 25).

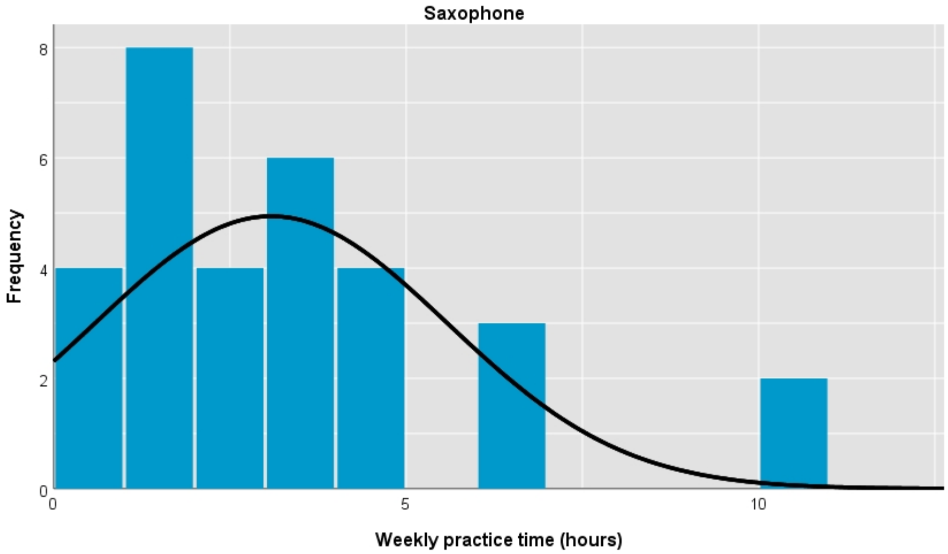

Supplementary Figure 20. Distribution of the weekly practice time, saxophone (n = 31).

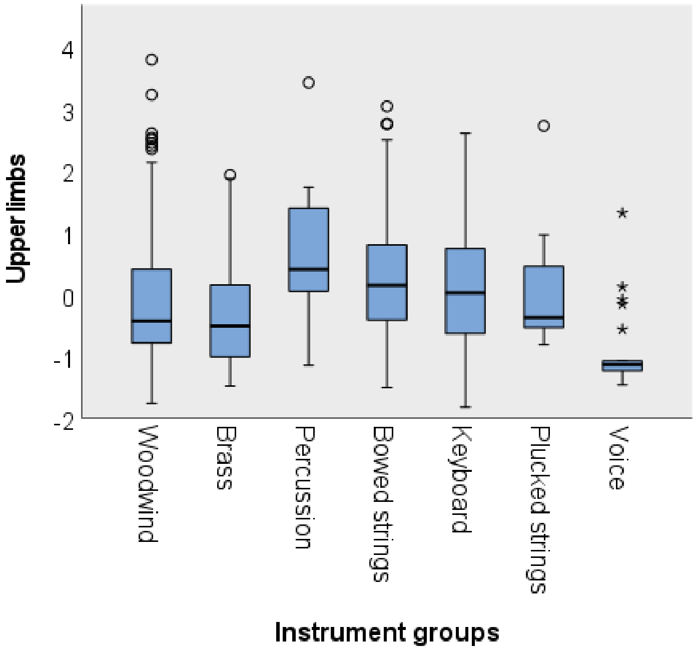

Supplementary Figure 21. Boxplots for z-standardized frequency of PRP in hands, fingers, wrists, arms (scale “upper limbs”) in different instrument groups (*n* = 549).

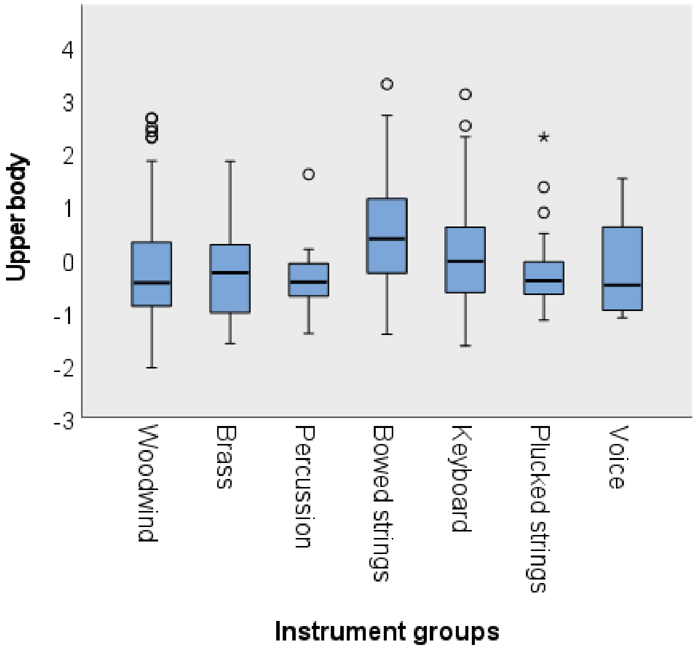

Supplementary Figure 22. Boxplots for z-standardized frequency of PRP in back, neck, shoulders, head (scale “upper body”) in different instrument groups (*n* = 549).

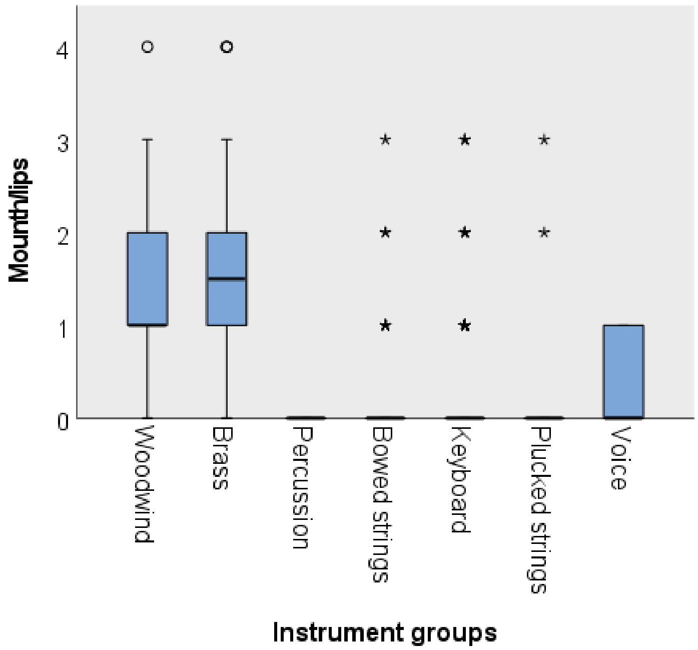

Supplementary Figure 23. Boxplots for frequency of PRP in mouth/lips in different instrument groups (*n* = 625).

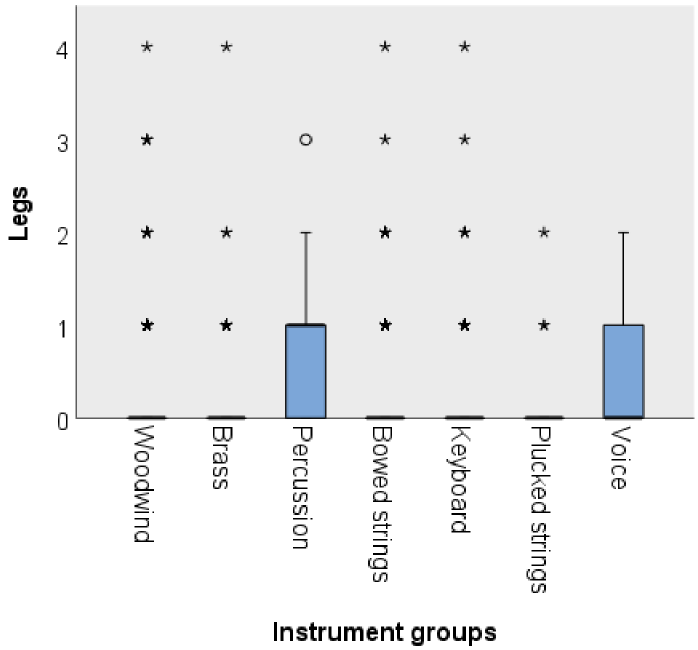

Supplementary Figure 24. Boxplots for frequency of PRP in legs in different instrument groups (*n* = 612).

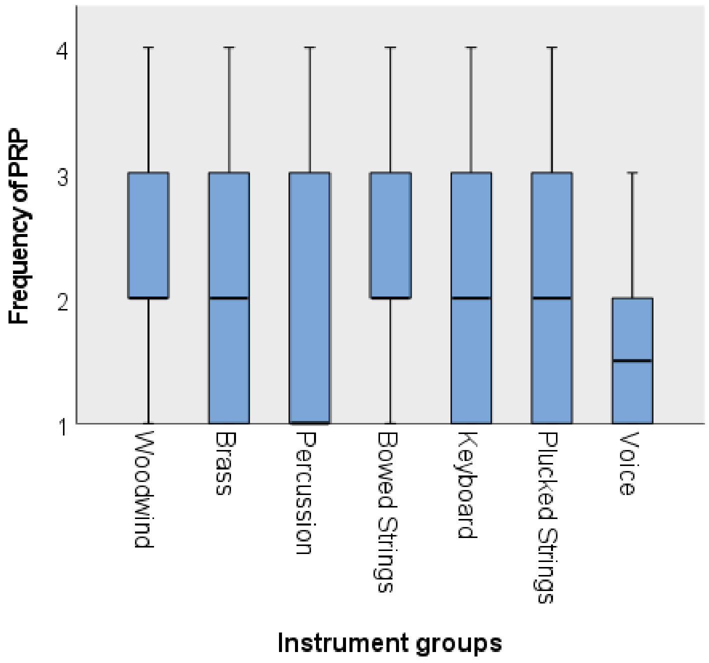

Supplementary Figure 25. Boxplots for frequency of PRP in different instrument groups (n = 820).

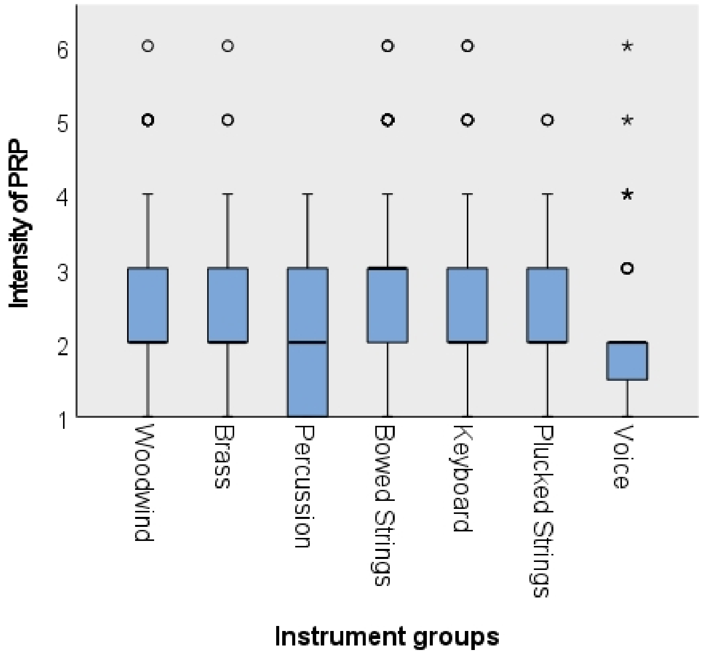

Supplementary Figure 26. Boxplots for intensity of PRP in different instrument groups (n = 743).

2 Supplementary Excerpt from the Questionnaire

Health (translation)

Has it ever happened that something hurt you while playing your instrument / while practicing or after playing?

☐ No, I have never had pain when making music / practicing.

☞ Please skip the following questions and continue with question 36.

☐ Yes, I have had pain when making music / practicing before.

☞ Please fill out the following table.

Body area	Always	Often	Frequently	Seldom	Never
Mouth / Lips	4	3	2	1	0
Neck and shoulder area	4	3	2	1	0
Arms	4	3	2	1	0
Hands	4	3	2	1	0
Wrist	4	3	2	1	0
Fingers	4	3	2	1	0
Legs	4	3	2	1	0
Back	4	3	2	1	0
Head	4	3	2	1	0
Other, namely: ____________________________	4	3	2	1	0

Do you have any other pain that has nothing to do with practicing?

☐ No ☐ Yes, that is: ____

If you have indicated pain while playing/practicing, how strong is the pain?

Very low					Very strong
1	2	3	4	5	6

Can you remember the first time you had pain while practicing?

☐ Always ☐ Already longer, for about _________ years now

☐ Not yet that long, since about _________ month(s)

Have you ever been to a doctor because of the pain while practicing?

☐ No ☐ Yes

What do you do if something hurts you while practicing?

(If true, you can also tick several items.)

☐ I take a break. ☐ I practice less.

☐ I relax, and I do it this way: ____

☐ I am doing something different: ____

Have you ever told anyone about the pain when you practice?

(If true, you can also tick several items.)

☐ My mother ☐ My father ☐ Friends ☐ Instrumental teacher

☐ Other persons, namely: ____

Do you feel that your pain is taken seriously when you practice?

☐ No ☐ Yes ☐ Partially

Do you do sports?

☐ No ☐ Yes, about _________ times per week.
